# H2A.Z acetylation by lincZNF337-AS1 via KAT5 implicated in the transcriptional misregulation in cancer signaling pathway in hepatocellular carcinoma

**DOI:** 10.1038/s41419-021-03895-2

**Published:** 2021-06-12

**Authors:** Yin Yuan, Wen Cao, Hongbing Zhou, Haixin Qian, Honggang Wang

**Affiliations:** 1 The Department of Hepatobiliary Surgery of Hospital Affiliated 5 to Nantong University(Taizhou People’s Hospital), Taizhou, Jiangsu Province China; 2 The Department of Liver Disease of Hospital Affiliated 5 to Nantong University(Taizhou People’s Hospital), Taizhou, Jiangsu Province China; 3 Department of General Surgery, The First Affiliated Hospital of Soochow University, Suzhou, Jiangsu Province China; 4 The Department of General Surgery of Hospital Affiliated 5 to Nantong University(Taizhou People’s Hospital), Taizhou, Jiangsu Province China

**Keywords:** Oncogenes, Post-translational modifications, Prognostic markers

## Abstract

In eukaryotes, histones and their variants are essential for chromatin structure and function; both play important roles in the regulation of gene transcription, as well as the development of tumors. We aimed to explore the genomics data of hepatocellular carcinoma (HCC), combined with literature analysis, in terms of the histone variant H2A.Z. Cell phenotype assay confirmed the effect of H2A.Z on the proliferation, metastasis, apoptosis, and cell cycle of HCC cells. H2A.Z was shown to function via the tumor dysregulation signaling pathway, with BCL6 as its interacting protein. In addition, the acetylation level of H2A.Z was higher in HCC and was related to tumor formation. We found the acetylation of H2A.Z to be related to and regulated by lincZNF337-AS1. LincZNF337-AS1 was found to bind to H2A.Z and KAT5 at different sites, promoting the acetylation of H2A.Z through KAT5. We concluded that, in HCC, H2A.Z is an oncogene, whose acetylation promotes the transcription of downstream genes, and is regulated by lincZNF331-AS1.

## Background

Hepatocellular carcinoma (HCC) is the most common cancer among adults and accounts for approximately 740,000 deaths worldwide [1]. It is highly prevalent in China, where new cases of HCC and related deaths are more than half of its global number [2]. In eukaryotes, histones are the most basic components of nucleosomes that are necessary for the structure and function of chromatin [3]. Histone variants are variants of conventional histone proteins that regulate chromatin structure and related biological processes by replacing the conventional histone proteins at specific locations of the chromatin or in specific biological events. Histone variants play an important role in the formation and maintenance of higher chromatin structures in eukaryotes and in the epigenetic mechanisms of cell programming and reprogramming [4, 5]. With the development of proteomics, histone variants have been found to play an important role in the regulation of gene transcription as well [6–10]. Histones and their variants also play crucial roles in different tumors. In a study of breast cancer, H4G was reported to promote the proliferation of breast cancer cells [11]. In malignant melanoma, mH2A was proven as a tumor suppressor gene in melanoma cells, with CDK8 possibly being the downstream target gene [12]. This study aimed to investigate whether HCC could as well be associated with a histone or histone variant that is critical for the development of tumor, and if so, what would be the underlying mechanism.

## Results

### RNA sequencing (RNA-seq) results and identification of target genes

**Fig. 1:**
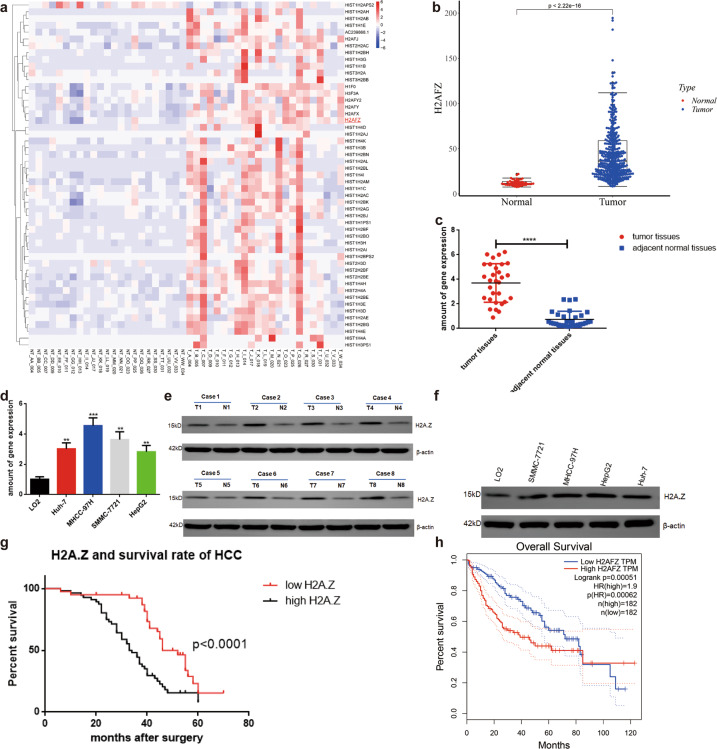
H2A.Z is highly expressed in HCC tissues and cells and is associated with the prognosis in patients with HCC. **a** Heat maps of histones and variants in 23 HCC and paired paracancer tissues analyzed using RNA-seq(Software: R pheatmap, Algorithm: ward2). **b** H2A.Z transcript between HCC tissues and normal liver tissues was analyzed in the publicly accessible samples(TCGA http://gepia.cancer-pku.cn/; *T* = 375, *N* = 50)(Software: beeswarm, R package). **c** Scatter plots comparing H2A.Z expression in HCC samples and normal liver tissue samples detected by qPCR(Student’s *t*-test used in Fig **c**, *n* = 30 in each group). **d** The expression levels of H2A.Z in human HCC cells were detected by qRT-PCR. **e** Expression levels of H2A.Z protein were detected in 8 matched HCC samples by western blot (*N*, normal tumor-adjacent tissue; *T*, tumor tissue). **f** Expression levels of H2A.Z protein were detected in HCC cell lines by western blot. **g** Kaplan-Meier survival curves illustrating the overall survival and disease-free survival of patients with HCC associated with H2A.Z expression in 96 cases. **h** The negative correlation between H2A.Z expression and overall survival was analyzed on the basis of TCGA data in patients with HCC(Software: survival, R package). The data are expressed in terms of mean ± SD(Student’s *t*-test used in Fig **d** and Kaplan-Meier test used in Fig **g**; **P < 0.01; ***P < 0.001; ****P < 0.0001; *n* = 3 in each group).

A previous search in the GEO (Group on Earth Observations) database yielded 23 pairs of raw data (not transcripts) (GSE138485). To verify the reliability of the data, we explored three pairs of genes from HCC cells and paracancerous tissues (BioProject: PRJNA642330) (Fig. S1C–D). A cumulative distribution curve, based on the analysis of the above two data, indicated the difference to be highly consistent [13] (Fig. S1A–B, E). We analyzed all the histone and histone variant genes using genomics data (GSE 138485) and found H2A.Z, known to be associated with breast cancer and prostate cancer, to be significantly expressed in HCC tissues [14, 15] (Fig. 1A). Online analysis based on TCGA indicated significantly higher H2A.Z levels in HCC tissues than in normal liver tissues (Fig. 1B). Furthermore, H2A.Z levels were determined in 30 pairs of HCC tissues and matched non-cancer tissues, and 96 cases of frozen tumor tissue samples collected from patients at the Department of Hepatobiliary Surgery of the Fifth Affiliated Hospital of Nantong University(Taizhou People’s Hospital) (Taizhou, Jiangsu, China). The H2A.Z levels were significantly increased in primary HCC tissues than in their matched adjacent normal tissues (Fig. 1C, E). Moreover, H2A.Z levels in HCC cell lines were also increased compared to those in the normal human liver cell line (Fig. 1D, F). Cox risk model was used to analyze the clinical data and prognosis of patients with HCC; H2A.Z expression, TNM stage, and vascular invasion were found to be independent clinical predictors of overall survival rate and recurrence rate in HCC (Table 1). Importantly, high H2A.Z levels in HCC significantly predicted poor outcomes in patients with HCC (Fig. 1G). In addition, online analysis based on TCGA data (http://gepia.cancer-pku.cn/) showed H2A.Z levels to be negatively correlated with the overall survival of patients with HCC (Fig. 1H).

**Table 1:** Cox regression analyses of factors associated with OS and TTR of HCC patients (*n* = 96).

Variables	Overall survival		Time to recurrence	
	Hazard ratio (95%CI)	*P*-value	Hazard ratio (95%CI)	*P*-value
Sex (female vs. male)	1.011 (0.987–1.034)	0.371	1.014 (0.991–1.038)	0.226
Age, Y (≤51 vs. >51)	1.671 (0.571–1.504)	0.759	0.797 (0.492–1.294)	0.359
Tumor differentiation (III–IV vs. I–II)	2.625 (0.629–4.440)	0.303	1.626 (0.683–3.870)	0.272
AFP (ng/mL; >20 vs. ≤20)	1.518 (0.762–3.023)	0.235	1.019 (0.529–1.965)	0.955
HbsAg (negative vs. positive)	1.055 (0.443–2.513)	0.904	1.099 (0.438–2.757)	0.841
Liver cirrhosis (no vs. yes)	0.990 (0.463–2.114)	0.978	1.002 (0.439–2.288)	0.996
Tumor number (multiple vs. single)	1.056 (0.405–2.775)	0.911	0.817 (0.323–2.064)	0.669
Tumor encapsulation (yes vs.no)	1.131 (0.478–2.667)	0.779	1.491 (0.646–3.474)	0.353
Vascular invasion (yes vs.no)	2.625 (1.483–2.625)	0.001**	2.027 (1.152–3.570)	0.014*
TNM stage (III–IV vs. I–II)	1.958 (1.079–3.553)	0.027*	1.815 (1.024–3.217)	0.041*
H2A.Z (high vs. low)	2.917 (1.456–5.845)	0.003**	2.377 (1.250–4.522)	0.008**

*H2A.Z* H2A histone family member Z, *TNM* tumor, node, and metastasis, *AFP* alpha-fetoprotein.

*p < 0.05, **p < 0.01.

### H2A.Z promoted the proliferation of HCC cell, reduced their apoptosis, and aided invasion and metastasis

**Fig. 2:**
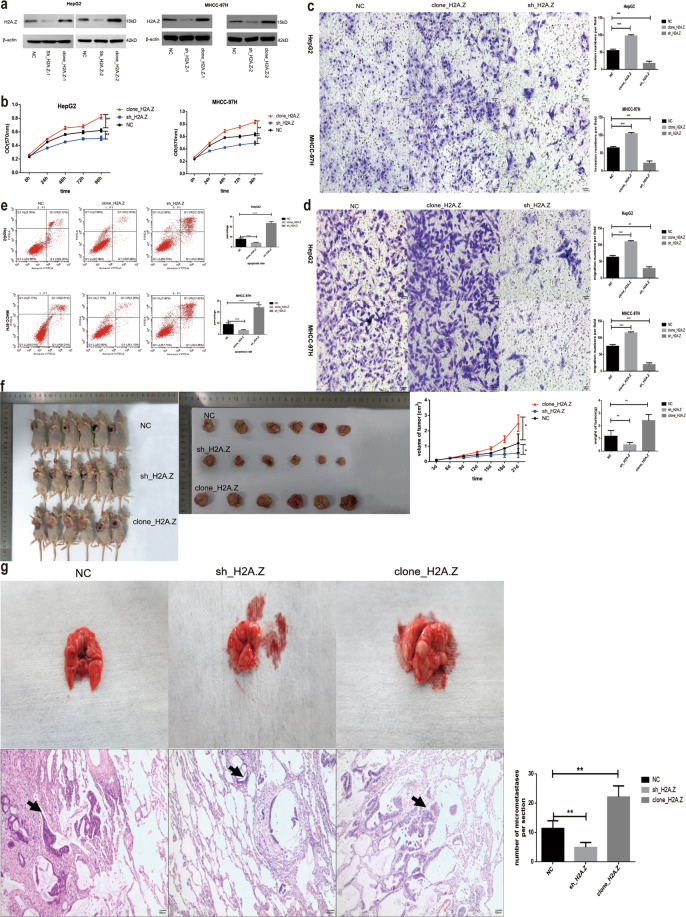
Effect of H2A.Z on cell growth. **a** Identification of the H2A.Z transfection effect detected by western blot. **b** The proliferation capability of cells was detected by MTT assays. **c**, **d** Transwell assay was adopted to test cell migration and invasion under different contexts(Scale bars: 50 μm). **e** Flow cytometry was utilized to test the apoptotic cells. **f** A subcutaneous tumor model in nude mice was used to test the tumor-forming ability of cell lines with different treatments. **g** A mouse lung metastasis model was used to detect cell metastasis under different contexts (The black arrow marks the metastases) (Scale bars: 100 μm). The data are expressed in terms of mean ± SD (Student’s *t*-test used in Fig **b**–**g**; *P < 0.05; **P < 0.01; ***P < 0.001. ****P < 0.0001; *n* = 3 in each group).

Since H2A.Z was overexpressed in HCC, knockdown and gain-of-function approaches were employed to determine the role of H2A.Z in HCC cells. Stable H2A.Z-knockdown (sh_H2A.Z-1 and sh_H2A.Z-2) and -overexpression (clone_H2A.Z-1 and clone_H2A.Z-2) HepG2 and MHCC-97H cell lines were established (Fig. 2A). Based on WB results, the HepG2 cell line was selected for sh_H2A.Z-1 and clone_H2A.Z-2, and the MHCC-97H cell line was selected for sh_H2A.Z-1 and clone_H2A.Z-1 in experiments investigating cell tumorigenic properties. Their proliferation ability was determined by the MTT assay. As shown in Fig. 2B, H2A.Z knockdown decreased the proliferation of HCC cells while its overexpression increased the same. Transwell cells without matrix gel were used to compare the difference in migration ability of the stable transgenic cells. Results showed the cell migration ability to be significantly increased upon overexpression of H2A.Z and decreased upon the interference of H2A.Z (Fig. 2D). The invasion ability of cells was significantly increased upon H2A.Z overexpression; however, it was significantly reduced after the interference of H2A.Z (Fig. 2C). Apoptosis increased after H2A.Z silencing and cell cycle progression slowed at the G1 phase, whereas apoptosis was significantly reduced upon H2A.Z overexpression (Fig. 2E and Fig. S2). Eighteen nude mice were randomly divided into three groups of six mice each. NC, clone_H2A.Z, and sh_H2A.Z with stable expression in HCC cell lines were injected into the armpits of nude mice to examine tumorigenicity. Three weeks later, the nude mice were sacrificed, and tumor samples removed surgically. Compared to the blank plasmid group, the H2A.Z gene-overexpression group had significantly larger tumors while the silenced group had significantly smaller tumors (Fig. 2F). In a mouse model of metastasis, no nodule was observed on the lung surface in the control and sh_H2A.Z groups, whereas they were observed on the lung surface in the clone_H2A.Z group. After HE staining of lung tissue sections, the number of metastatic nodules was analyzed under a 100× light microscope; results showed the number to have decreased in the sh_H2A.Z group and increased in the clone_H2A.Z group. H2A.Z enhanced the tumor metastasis ability in vivo (Fig. 2G).

### H2A.Z influenced the proliferation, apoptosis, cell cycle, and metastasis of HCC cells through transcriptional misregulation in the cancer signaling pathway

**Fig. 3:**
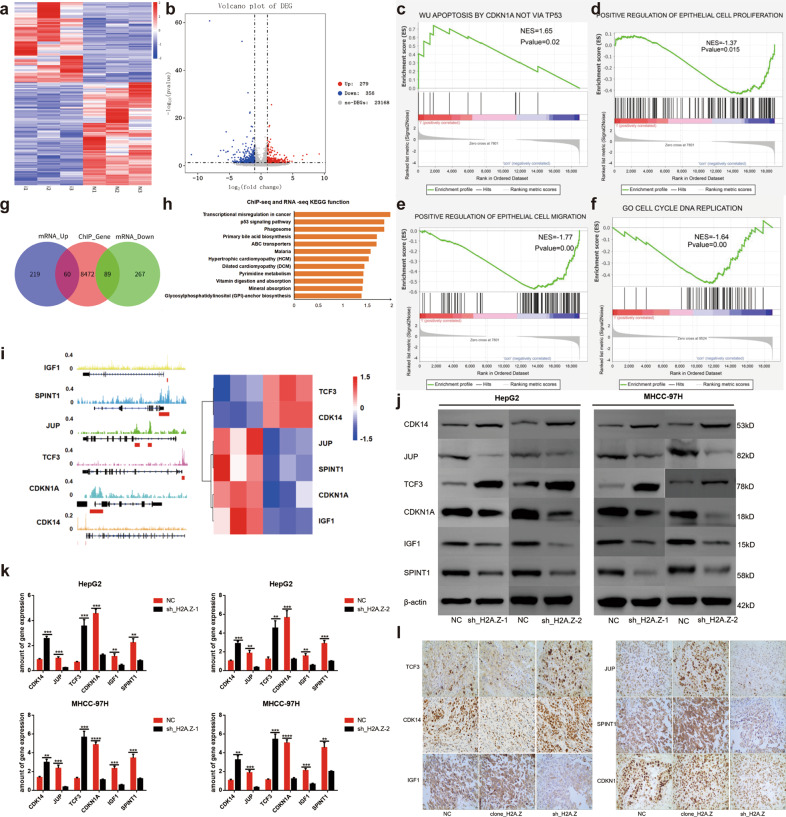
H2A.Z plays a pro-tumor role through transcriptional misregulation in cancer signaling pathways. **a** Heat map of whole-gene transcriptomic changes detected by RNA-seq in HepG2 cells that knockdown H2A.Z expression(Software: R pheatmap, R package). **b** Volcanogram of changes at the whole-gene transcriptomic level in HepG2 cells that knockdown H2A.Z expression(Software: ggplot2, R package). **c**–**f** Enrichment of pathway in GSEA analysis of genes altered as described above (Software: GESA). **g** Venn diagram depicting the overlapping variation genes in RNA-seq and Chip-seq of H2A.Z(Software: Venn Diagram, R package). **h** KEGG pathway analysis was used to analyze the combined analysis results showed that H2A.Z might be associated with transcriptional misregulation in cancer signaling pathway in HCC cells (Software: ggplot2, R package). **i** IGV diagram of the downstream target gene of H2A.Z in the combined analysis data. **j**, **k** Changes in the expression of target genes upon H2A.Z were knockdown in HCC cells detected by qPCR and western blot. **l** Immunohistochemical analysis of target proteins in tumors in nude mice from different groups(Scale bars: 20 μm). The data are expressed in terms of mean ± SD (Student’s *t*-test used in Fig **k**; **P < 0.01; ***P < 0.001. ****P < 0.0001; *n* = 3 in each group).

To gain insight into the mechanism by which H2A.Z regulates neoplastic characteristics of HCC cells, we performed RNA-seq using HCC cells (HepG2) with normal expression or knockdown of H2A.Z (GEO: PRJNA668278). RNA-seq showed the ratio of the number of upregulated to downregulated genes, due to H2A.Z knockdown in HepG2 cells, to be 279:356 (Fig. 3A–B). Subsequently, we analyzed the relevant tumorigenic pathways in tumor cells, as a whole, from the gene set enrichment analysis (GSEA) database. The latter revealed significant enrichment of apoptosis by CDKN1A, not via TP53 pathway genes, in H2A.Z-knockdown HepG2 cells (Fig. 3C). Regarding cell proliferation and migration phenotypes, GSEA analysis showed the genes to be mainly enriched in positive regulation of epithelial cell proliferation and epithelial cell migration pathways (Fig. 3D–E). In addition, genes were mainly enriched in the C5_go_cell_cycle_DNA_replication pathway in the cell cycle phenotype (Fig. 3F). In line with the observations mentioned above, the analyses confirmed H2A.Z to be closely related to proliferation, apoptosis, cell cycle, migration, and metastasis of HCC cells.

To further identify the potential downstream targets of H2A.Z, we jointly analyzed the RNA-seq data and ChIP-seq data of H2A.Z in the HepG2 cell line. The ChIP-seq data of H2A.Z was derived from the ENCODE database (GSM733774) (Table S9). Results showed 149 overlapping genes in the two groups including 60 co-upregulated genes and 89 co-downregulated genes (Fig. 3G). KEGG functional enrichment analysis of overlapping genes indicated the genes to be mainly enriched in the transcriptional misregulation in the cancer signaling pathway (Fig. 3H). Further analysis of gene enrichment of the transcriptional misregulation in cancer signaling pathway revealed *TCF3* (transcription factor 3), *CDK14* (cyclin-dependent kinase 14), *JUP* (junction plakoglobin), *SPINT1* (serine peptidase inhibitor, Kunitz type 1), *CDKN1A* (cyclin-dependent kinase inhibitor 1A, p21), and *IGF1* (insulin-like growth factor 1) as downstream target genes that influence the phenotype of tumor cells (Fig. 3I, Fig. S3A). Next, we used ChIP-qPCR to demonstrate that H2A.Z can bind to the promoter region of the above six downstream genes (Fig. S3B, Table S6, S9). Finally, we verified the relevant changes in HCC cell lines and nude mouse tumors and found them to be consistent with those obtained from the combined analysis (Fig. 3J-L).

### BCL6 was identified as the main interacting protein of H2A.Z in HCC and BTB and PHA03247 as the main binding domains

**Fig. 4:**
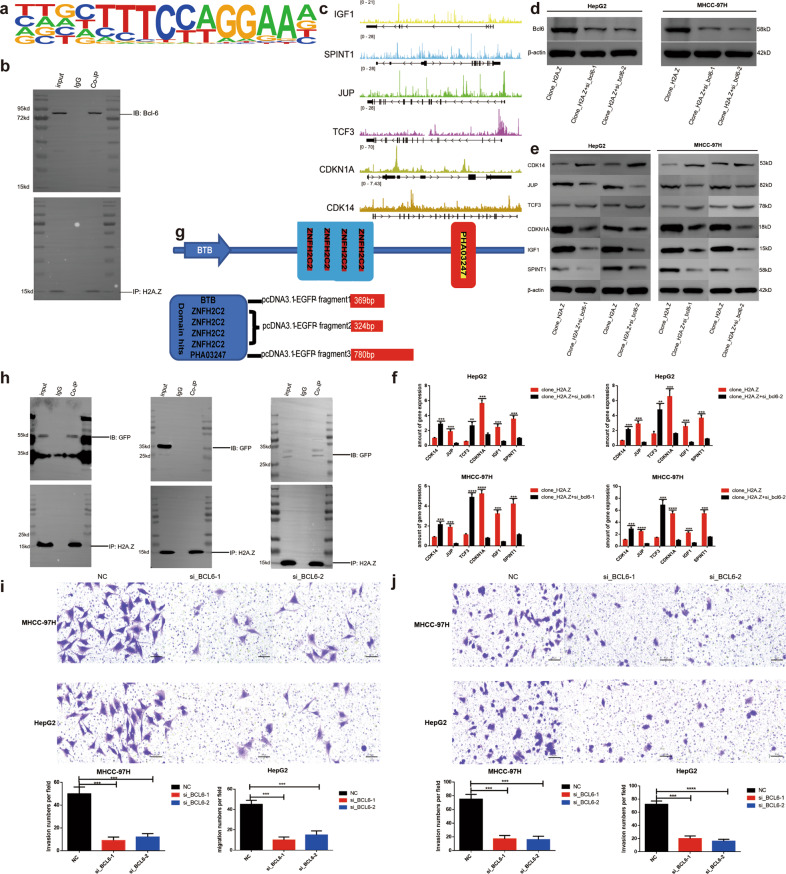
BCL6 is the main interaction protein of H2A.Z in HCC, and the protein domains binding to H2A.Z are BTB and PHA03247. **a** Motif analysis of H2A.Z and BCL6 in chip-seq of H2A.Z. **b** Co-IP analysis of H2A.Z and BCL6 in HepG2 cell. **c** IGV diagram of the downstream target gene of H2A.Z in the combined analysis data of H2A.Z RNA-seq and BCL6 chip-seq. **d** Validation of BCL6 gene knockout effect detected by western blot. **e**, **f** QRT-PCR, and western blot verified the changes of downstream target genes after BCL6 knockdown in H2A.Z overexpressed HCC cell lines. **g** Schematic diagram of the BCL6 protein domain(https://www.uniprot.org/). **h** The interaction of BCL6 protein regions with H2A.Z in HEK293T cells was confirmed by a Co-IP assay, which showed the protein domains binding to H2A.Z are BTB and PHA03247. **i**, **j** Transwell assay was adopted to test cell migration and invasion under different contexts. The data are expressed in terms of mean ± SD (Student’s *t*-test used in Fig **h**, **i** and **j**; **P < 0.01; ***P < 0.001; ****P < 0.0001; *n* = 3 in each group).

We analyzed the motif data in ChIP-seq of H2A.Z, and the results suggested BCL6 as the most probable interacting protein of H2A.Z (Fig. 4A, Table S8). We subsequently demonstrated an interaction between the two proteins in HepG2 cell lines (Fig. 4B). In order to address how BCL6 could be involved in HCC, we analyzed the ChIP-seq data of BCL6 (GSE96359) (Table S9) and compared it with RNA-seq data of H2A.Z. Results showed the binding data of six downstream H2A.Z target genes to be consistent. ChIP-qPCR further confirmed that BCL6 could also bind to the promoter region of the above six downstream genes (Fig. S4A, Table S7, S9). These results further confirmed the interaction of BCL6 with H2A.Z, which in turn affected the downstream gene transcription (Fig. 4C). Finally, knockout verification was conducted in HCC cell lines(Fig. 4D). Once BCL6 was knocked out in stable cell lines with overexpression of H2A.Z, changes in downstream target genes were consistent as per omics analysis (Fig. 4E, F). Since H2A.Z is a small (15 kD) protein and BCL6 is a relatively larger (78 kD) protein, the binding site for interaction of H2A.Z and BCL6 would be worth exploring. First, we identified the domain hits of BCL6 on the protein website: https://www.uniprot.org/, including mainly BTB, four-segment znfH2C2, and PHA03247 (Fig. 4G). Subsequently, the coding sequences for the three amino acid sequences, labeled with eGFP, were inserted into the pcDNA3.1 plasmid and co-transfected in HEK293T cells along with the H2A.Z overexpression vector. Finally, Co-IP experiments revealed H2A.Z to mainly bind to the BTB and PHA03247 domains of BCL6 protein (Fig. 4H). Further, silencing of BCL6 in HCC cell lines decreased the invasion and migration abilities of tumor cells (Fig. 4I–J).

### Acetylation level of H2A.Z was significantly increased in HCC

**Fig. 5:**
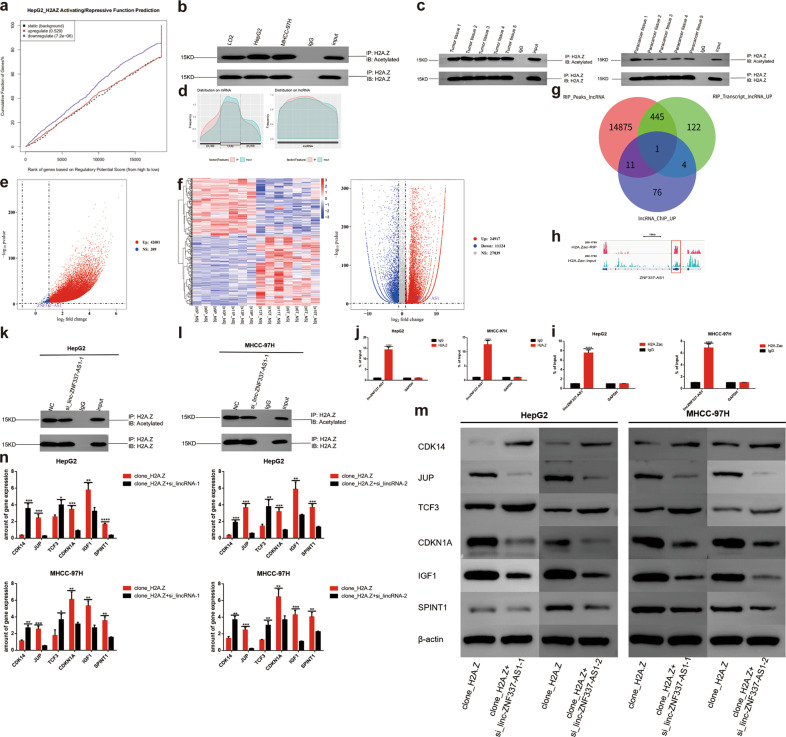
LincZNF337-AS1 binds to H2A.Z and H2A.Zac, and regulates the acetylation of H2A.Z. **a** H2A.Z mainly promoted the transcription of downstream genes(Software: BETA, algorithm: basic-k BSF-df 0.05). **b** The acetylation level of H2A.Z was detected by Co-IP in HCC cell lines. **c** The acetylation level of H2A.Z was detected by Co-IP in HCC tissues. **d** RIP with H2A.Zac antibody in HepG2 cells and sequencing. **e** Volcanograms of lincRNA identified by RIP-seq(Software: ggplot2, R package). **f** Heat maps and volcanograms of the seven pairs of HCC and paired paracancer tissues analyzed by RNA-seq(Software: R pheatmap, R package). **g** Venn diagram of RIP-seq and lincRNA highly expressed in tumors in RNA-seq. **h** IGV diagram of lincZNF337-AS1. **i**–**j** Identification of lincZNF337-AS1 binding to H2A.Z and H2A.Zac by RIP-qPCR. **k**–**l** LincZNF337-AS1 regulates the acetylation of HCC, the level of acetylation was measured by Co-IP. **m**, **n** QRT-PCR, and western blot verified the changes of downstream target genes after lincZNF337-AS1 knockdown in H2A.Z overexpressed HCC cell lines. The data are expressed in terms of mean ± SD (Student’s *t*-test used in Fig **i**, **j**, and **n**; **P < 0.01; ***P < 0.001; ****P < 0.0001; *n* = 3 in each group).

Combined analysis of ChIP-seq and RNA-seq of H2A.Z showed the downregulated genes of the H2A.Z-knockdown group to be significantly correlated with ChIP-seq data; therefore, H2A.Z was considered to mainly play an activating role (Fig. 5A). Epigenetic studies of histones have shown their post-transcriptional modifications to be diverse, including acetylation, methylation, ubiquitination, phosphorylation, and SUMOylation, with acetylation specifically known to alter chromatin aggregation, thereby promoting the transcription of downstream genes [16–19]. We hypothesized high acetylation of H2A.Z to promote the transcription of downstream genes in HCC. We determined the acetylation level of H2A.Z in HCC cell lines and tissue samples and found it to be significantly increased in both (Fig. 5B–C).

### LincZNF337-AS1 bound to H2A.Z and H2A.Zac regulated the acetylation of H2A.Z and affected the transcription of downstream target genes

With the development of lincRNA research, increasing evidence has shown lincRNA to play an important role in post-transcriptional modification of proteins, in particular, epigenetic modification of histones [20–23]. Whether lincRNA is involved in the acetylation of H2A.Z was considered worth exploring. Therefore, we performed a RIP experiment with acetylated H2A.Z protein and sequenced the lincRNA data associated with it (GEO: PRJNA668278). Since there was no antibody to cover all the acetylation sites on H2A.Z, we chose an antibody from Abcam (No. Ab232908) that contained multiple common sites. Considering the high expression of H2A.Z in HCC, we speculated lincRNA expression, combined with H2A.Z, to be upregulated. We analyzed a set of omics data for HCC and paired normal tissue in the GEO database (GSE101728) and combined them with RIP-seq data to obtain a target lincRNA, namely, lincZNF337-AS1 (Fig. 5D–H). RIP-qPCR confirmed that lincZNF337-AS1 indeed bound to H2A.Zac. In addition, lincZNF337-AS1 also bound to H2A.Z (Fig. 5I–J). After lincZNF337-AS1 was knocked out (Fig. S4B–C), the acetylation level of H2A.Z in cell lines decreased (Fig. 5K–L), indicating that lincZNF337-AS1 regulates the acetylation of H2A.Z. Moreover, after lincZNF337-AS1 was knocked out in H2A.Z-overexpressed stable cell lines (Fig. S4D–E), all the downstream target genes of H2A.Z were altered, which further indicated lincZNF337-AS1-binding to H2A.Z to affect the transcription of downstream genes (Fig. 5M, N).

**Fig. 6:**
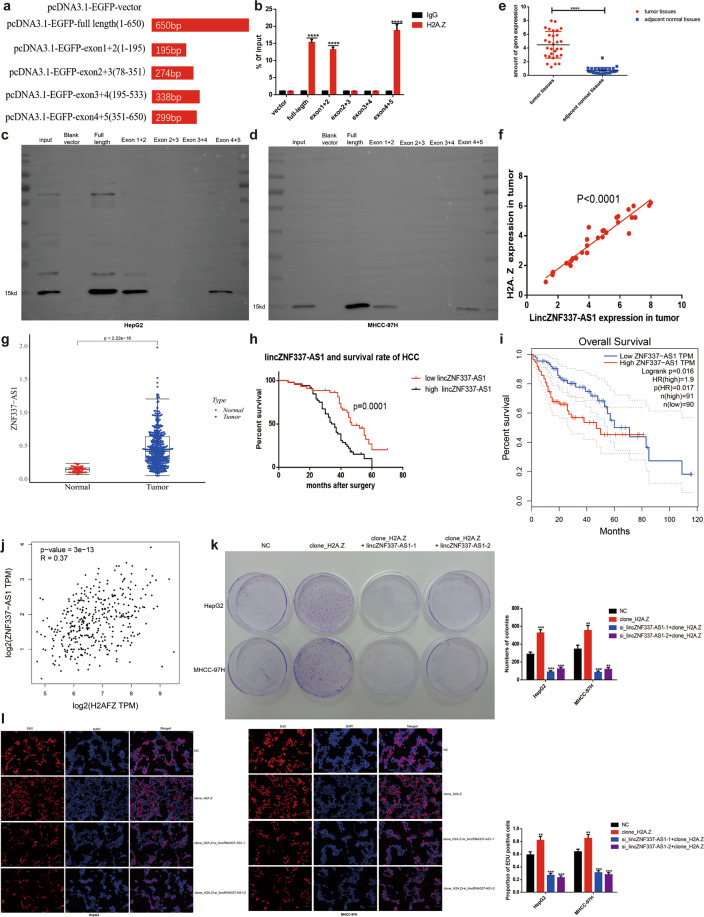
Identification of lincZNF337-AS1 binding sites to H2A.Z, identification of lincZNF337-AS1 expression levels in HCC, and relationship with prognosis of HCC patients. **a** Schematic diagram of lincZNF337-AS1 full-length and truncated fragments. **b** The interaction of lincZNF337-AS1 truncated fragments with H2A.Z in HEK293T cells was verified by a RIP-qPCR assay. **c**, **d** RNA-pulldown assay verifies the binding site of lincZNF337-AS1 to H2A.Z in HCC cells. **e** Scatter plots comparing lincZNF337-AS1 expression in HCC samples and normal liver tissue samples detected by qPCR (Student’s t-test used in Fig **e**, *n* = 30 in each group). **f** The positive correlation between lincZNF337-AS1 and H2A.Z expression was analyzed on the basis of data of 30 HCC samples. **g** LincZNF337-AS1 transcript between HCC tissues and normal paracancer tissues was analyzed in the publicly accessible samples(TCGA http://gepia.cancer-pku.cn/, *T* = 375; *N* = 50, Software: beeswarm, R package). **h** Kaplan–Meier survival curves illustrating the overall survival and disease-free survival of patients with HCC associated with lincZNF337-AS1 expression in 96 cases. **i** The negative correlation between lincZNF337-AS1 expression and overall survival was analyzed on the basis of TCGA data in patients with HCC(Software: survival, R package). **j** The positive correlation between lincZNF337-AS1 and H2A.Z expression was analyzed on the basis of TCGA data in patients with HCC(Software: ggplot2, R package). **k**, **l** The proliferation capability of cells was detected by colony formation assay and EdU assay (scale bar = 50 μm). The data are expressed in terms of mean ± SD (Student’s *t*-test used in **b**, **k**, and **l**; Kaplan-Meier test used in **g**; Linear regression test used in **j**; ****P < 0.0001; *n* = 3 in each group).

To predict the region of lincZNF337-AS1 responsible for interacting with H2A.Z, a series of lincZNF337-AS1 truncations were analyzed, and probabilities of their interacting with H2A.Z predicted (Fig. 6A). The final exon-truncated fragment sequences were loaded into a vector and co-transfected into HEK293T cells with the H2A.Z-overexpression vector. The experimental results from the RIP assay, using the H2A.Z antibody, confirmed the interaction between H2A.Z and lincZNF337-AS1 at two sites (Fig. 6B). Finally, the truncated sequence was transcribed into RNA in vitro (Fig. S5A-B, Table S5), and the binding sites of lincZNF337-AS1 to H2A.Z were reverse-verified using the RNA-pulldown method (Fig. 6C–D).

### LincZNF337-AS1 was highly expressed in HCC and was associated with H2A.Z expression; high lincZNF337-AS1 expression correlated with poor outcome in patients with HCC

To further define the role of lincZNF337-AS1 clinically and to verify its correlation with H2A.Z in clinical samples, we measured H2A.Z gene expression in 30 matched tissues using qPCR. Results showed lincZNF337-AS1 to be highly expressed in HCC (Fig. 6E), and the expression to be positively correlated with H2A.Z level (Fig. 6F). Online analysis based on TCGA data (http://gepia.cancer-pku.cn/) showed high expression of lincZNF337-AS1 in HCC(Fig. 6G). In the 96 clinical HCC specimens, a high lincZNF337-AS1 level significantly predicted a poor outcome in patients with HCC (Fig. 6H). In addition, the lincZNF337-AS1 level was to be positively correlated with H2A.Z level while the lincZNF337-AS1 level was negatively correlated with the overall survival of patients with HCC (Fig. 6I, J). These data indicated the lincZNF337-AS1/H2A.Z compound to be strongly correlated with HCC tumor biology, along with poor outcomes in patients with HCC. However, it was unclear whether lincZNF337-AS1 affects the tumorigenic properties of HCC cells. A comparison of parental cells, H2A.Z-overexpressed cells, and lincZNF337-AS1 knockdown cells revealed that the cloning ability (Fig. 6K) and proliferation ability (Fig. 6L) of tumor cells decreased after lincZNF337-AS1 knockdown, likely due to the changes in the expression of the above-mentioned key downstream genes.

### LincZNF337-AS1 promoted acetylation of H2A.Z through KAT5

**Fig. 7:**
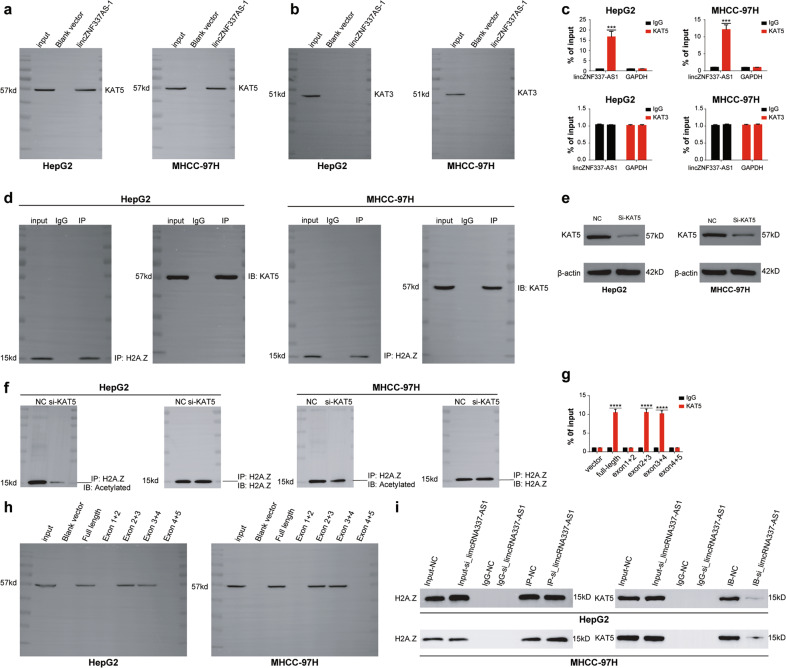
LincZNF337-AS1 promotes acetylation of H2A.Z through KAT5. **a**–**b** RNA-pulldown assay verifies lincZNF337-AS1 binding to KAT5, not KAT3. **c** RIP-qPCR assay verifies lincZNF337-AS1 binding to KAT5, not KAT3. **d** Co-IP assay identifies the interaction between H2A.Z and KAT5 in HCC cell lines. **e** Identification of KAT5 gene knockdown effect by western blot. **f**, **i** The effect of knockdown KAT5 and lincZNF337-AS1 on H2A.Z acetylation detected by Co-IP assay. **h** RNA-pulldown assay verifies the binding site of lincZNF337-AS1 to KAT5 in HepG2 and MHCC-97H cell lines. The data are expressed in terms of mean ± SD (Student’s *t*-test used in Fig **c** and **g**; ****P < 0.0001; *n* = 3 in each group).

Dozens of acetyltransferases have been identified to date. However, in eukaryotes and mammalian cells, acetyltransferases involved in the acetylation of H2A, and its variants mainly include KAT1, KAT3, KAT5, and KAT6, with KAT3 and KAT5 being the most common [24–28]. In order to check whether acetyltransferases could be involved in the process of H2A.Z acetylation regulated by lincZNF337-AS1, we first conducted RNA-pulldown and RIP-qPCR experiments to detect the binding of lincZNF337-AS1 with KAT3 and KAT5; results showed lincZNF337-AS1 to be bound to KAT5, though not to KAT3 (Fig. 7A–C). Therefore, we believed that lincZNF337-AS1 and KAT5 together promote acetylation of H2A.Z. We further verified the interaction between H2A.Z and KAT5 using the Co-IP experiment (Fig. 7D). We knocked down KAT5 in HCC cell lines and detected acetylation levels of H2A.Z in the cells. Results showed that the acetylation level of H2A.Z decreases significantly after KAT5 knockdown (Fig. 7E–F). Finally, we used RNA-pulldown to identify the specific site of lincZNF337-AS1 binding to KAT5 and found the interaction between KAT5 and lincZNF337-AS1 at a site distinct from the binding site of H2A.Z (Fig. 7G–H). Moreover, when we knocked down the expression of lincZNF337-AS1 in HCC cell lines, the interaction between H2A.Z and KAT5 was significantly reduced (Fig. 7I).

## Discussion

In eukaryotic cells, nucleosomes in the nucleus, consisting of H2A-H2B dimers, (H3-H4)2 tetramers, the DNA wound around them, and the connective histone H1, play an important role in gene transcription [29–31]. H2 histones are the most active ones with maximum variants [29]. A large number of studies have shown histone variants to be involved in eukaryotic gene regulation [33, 32]. The significance of histone and its variants in gene regulation has particularly drawn attention [34–36] in relation to the occurrence and development of various tumors, including HCC.

To investigate the histones and their variants involved in HCC, we analyzed them based on the RNA-seq data of 23 pairs of HCC and paired paracancerous tissues; results showed the expression level of histone variant H2A.Z to be significantly upregulated in tumor tissues, hence suggesting its possible role in HCC. The histone variant H2A.Z was originally identified in 1980 in mouse L1210 cells [37], followed by the cloning of mammalian H2A.Z gene in 1990 (ref. [38]); it was found to be essential since the knockout mouse displayed an early embryonic lethal phenotype [39]. Previous studies have shown H2A.Z to be involved in gene transcription [19, 40–44]; like all typical histones, H2A.Z is also dynamically modified by various PTMs (including acetylation, methylation, phosphorylation, ubiquitination, and SUMOylation), which can change the chromatin state and affect downstream gene transcription [16, 19, 43, 45]. In addition, H2A.Z has been proven to be related to a variety of tumors, including breast cancer, prostate cancer, skin cancer, liver cancer, and HCC. H2A.Z is an oncogene that promotes the generation and development of tumors [14, 15, 46–49].

Whether H2A.Z is a tumor driver gene in HCC and the mechanisms involved remain unclear. Tang et al. [50]. believed that H2A.Z could be important for the development and progression of HCC and could be closely related to RNA splicing; however, their bioinformatic analysis yielded insufficient evidence to support this theory. Therefore, our study focused on the biological function and mechanism of H2A.Z in HCC.

We first verified H2A.Z in HCC tissues and HCC cell lines and found both gene and protein expression levels to be significantly increased in HCC. Regarding its effect on tumor cell phenotype, we found H2A.Z to promote proliferation and metastasis, reduce apoptosis, and accelerate the cell cycle of HCC cells. In order to explore the mechanism, RNA-seq was performed on the H2A.Z-knockdown cell line and untreated cell line; GSEA pathway analysis suggested the relevant pathways in the above tumor cell phenotypes. The combined analysis of RNA-seq and ChIP-seq for H2A.Z showed downstream gene regulation to mainly occur via the transcriptional misregulation in the cancer pathway, its target genes mainly including *TCF3 CDK14, JUP, SPINT1, CDKN1A*, and *IGF1*, each of which corresponded to different cell phenotypes. The ChIP-seq data of H2A.Z suggested BCL6 to possibly be the interaction protein of H2A.Z, and the interaction between BCL6 and H2A.Z proteins was verified to affect the transcription of downstream target genes. The specific binding site of BCL6 protein was also investigated, and the specific domain of BCL6 binding to H2A.Z determined.

In eukaryotic cells, posttranscriptional modification of histones and their variants is closely related to gene transcription, and H2A.Z acetylation is known to result in an open chromatin state that promotes downstream gene transcription [18, 19]. In addition, Liu et al. [51], using high-throughput computing technology, studied the modification of important histones in HCC and found that the acetylation level of H2A.Z was increased in HCC cell lines, which was related to the highly expressed genes in the HCC cell lines. In our study, RNA-seq and ChIP-seq analyses of H2A.Z showed it to mainly promote the transcription of downstream genes in HCC, thereby suggesting the acetylation levels of H2A.Z in HCC to possibly be increased. We verified and confirmed this speculation in HCC tissues and HCC cell lines.

Recent studies have shown that lincRNAs can bind to proteins and regulate their post-transcriptional modification [20, 23]. We conducted RIP-seq experiments to determine whether a lincRNA could be involved in the acetylation of H2A.Z, and eventually identified lincZNF337-AS1, based on the results of omics analysis. Cell function experiments including EdU assay and colony formation assay also demonstrated that lincZNF337-AS1 is an important regulator of HCC growth. A series of validation experiments showed lincZNF337-AS1 to combine with H2A.Z and H2A.Zac and promote the acetylation of H2A.Z. Since lincZNF337-AS1 was shown to interact with both H2A.Z and H2A.Zac, we considered it to regulate the acetylation of H2A.Z in a dynamic manner. In this study, we found that lincZNF337-AS1 binds H2A.Z and KAT5 at different sites, H2A.Z interacts with KAT5, and KAT5 promotes H2A.Z to complete its acetylation. In this mechanism, lincZNF337-AS1 plays a key bridging role. When we downregulated the expression of lincZNF337-AS1, the interaction between H2A.Z and KAT5 was significantly reduced. In addition, we found that acetylation of H2A.Z in HCC could change the chromatin status and promote the transcription of downstream genes, including *TCF3*, *CDK14*, *JUP*, *SPINT1*, *CDKN1A*, and *IGF1*, which are important regulatory molecules in the cancer misregulation pathway. Thus, this study has shown for the first time that lincZNF337-AS1 is involved in the regulation of H2A.Z acetylation by KAT5 to promote HCC growth. LincRNA has been a hot-spot molecule in recent years and has been reported to play an important role in transcriptional interference, inducing chromatin remodeling and histone modification, and regulation of protein activity and selective splicing [23]. The findings of this study expand the current knowledge about the biological functions of lincRNA and histones; however, it is unknown whether lincZNF337-AS1 can modulate other types of post-transcriptional modifications of H2A.Z by recruiting other epigenetic regulators. Our results, combined with our existing research, suggest that lincZNF337-AS1 likely plays an important role in the epigenetic regulation of H2A.Z; this should be further explored in future studies.

**Fig. 8:**
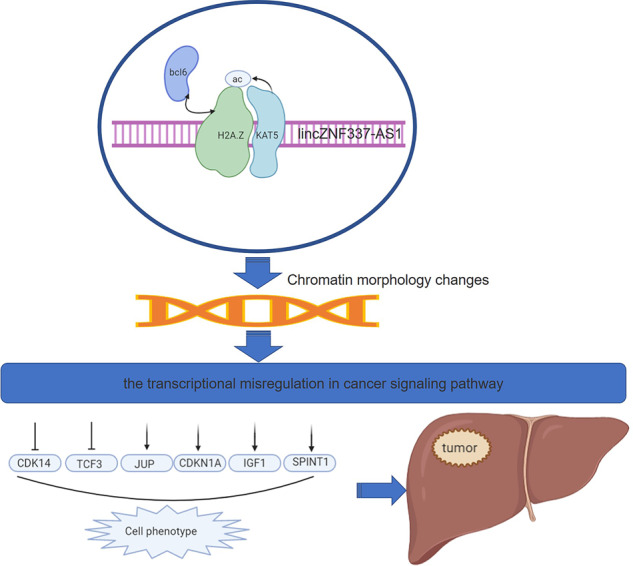
Schematic diagram of the action mechanism of H2A.Z in HCC. H2A.Z acetylation by lincZNF337-AS1 via KAT5 implicated in the transcriptional misregulation in cancer signaling pathway in HCC. Fig.S1 Bioinformatics data analysis. **a**–**b** Heat maps and volcanograms of 23 pairs of HCC and paired paracancer tissues analyzed using RNA-seq in the GEO database(Software: R pheatmap and ggplot2, R package). **c**–**d** Heat maps and volcanograms of the three pairs of HCC and paired paracancer tissues analyzed by RNA-seq(Software: R pheatmap and ggplot2, R package). **e** Graphical comparison of the two groups of gene transcriptome data analyzed using RNA-seq, smooth curve, indicating high consistency of data(Software: ggplot2, R package) (Fig. S2) Flow cytometry analysis was conducted for detecting the cell cycle. The data are expressed in terms of mean ± SD (Student’s *t*-test; *P < 0.05; **P < 0.01; *n* = 3 in each group) (Fig. S3) Verification of downstream target genes of H2A.Z. **a** Schematic diagram of transcriptional misregulation in the cancer signaling pathway, E47 synonymsTCF3; p21 synonyms CDKN1A; PFTK1 synonyms CDK14; plakoglobin synonyms JUP. **b** Chip-qPCR assay identifies the downstream target genes of H2A.Z. The data are expressed in terms of mean ± SD (Student’s *t*-test; ***P < 0.001; ****P < 0.0001; *n* = 3 in each group) (Fig. S4) Verification of downstream target genes of BCL6 and knockdown identification of lincZNF337-AS1 in vitro. **a** Chip-qPCR assay identifies the downstream target genes of H2A.Z. **b**–**e** Identification of lincZNF337-AS1 gene knockdown effect by qRT-PCR and ISH in HCC cell lines and H2A.Z overexpression HCC cell lines(Scale bars: 20 μm). The data are expressed in terms of mean ± SD (Student’s *t*-test; ***P < 0.001; ****P < 0.0001; *n* = 3 in each group) (Fig. S5) analysis of the in vitro transcription products. **a** Electrophoretic images of PCR products after plasmid amplification, the Maker strip size from top to bottom is in order: 5 K, 3 K, 2k, 1.5 K, 1 K, 750 bp, 500 bp, 250 bp, 100 bp. b Electrophoretogram of transcription products in vitro, Maker strip size from top to bottom: 2k 1 K 750 bp 500 bp 250 bp 100 bp (*n* = 3 in each group).

In conclusion, the histone variant H2A.Z is a proto-oncogene in HCC, which promotes the development of tumors and is related to the prognosis of patients. Bcl6 was confirmed to be the interacting protein of H2A.Z in HCC. Acetylation of H2A.Z is an important epigenetic modification in HCC; lincZNF337-AS1 binds to H2A.Z and KAT5 at different sites in order to promote this acetylation (Fig. 8).

## Materials and methods

### Patients and tissue samples

A total of 96 patients were enrolled in this study. Patients underwent curative resection for HCC at the Department of Hepatobiliary Surgery of Hospital Affiliated 5 to Nantong University(Taizhou People’s Hospital) (Taizhou, China) between January 2012 and December 2014. Patients did not receive any treatments before surgery. They were followed up after surgery until January 2019, with a median follow-up of 40 months (range, 6–70 months). During the follow-up, patients were monitored every 2 to 3 months. Computed tomography (CT) or magnetic resonance imaging (MRI) was performed when tumor recurrence was suspected. The patients’ clinicopathological characteristics are presented in Table 2. Clinical samples were collected from patients after signing the written informed consent form. The study protocol was approved by the Ethics Committee of the Hospital Affiliated 5 to Nantong University(Taizhou People’s Hospital). The tissue samples(including 30 matched tissue samples and 96 HCC tissue samples) were all from the biological sample bank of the Hospital Affiliated 5 to Nantong University (Taizhou People’s Hospital).

**Table 2:** Expression of H2A.Z by immunohistochemical and clinicopathologic features of HCC patients (*n* = 96).

Variables	Expression of H2A.Z low (40) high (56)		*p*
Sex			
Female	19	28	0.81
Male	21	28	
Age (years)			
≤51	19	25	0.78
>51	21	31	
Preoperative AFP(ng/mL)			
≤20	8	14	0.57
>20	32	41	
HBsAg			
Negative	16	19	0.54
Positive	24	37	
Liver cirrhosis			
No	15	15	0.20
Yes	25	44	
Tumor size (cm)			
≤5	22	21	0.09
>5	18	35	
Tumor number			
Single	28	42	0.59
Multiple	12	14	
Tumor encapsulation			
No	16	30	0.19
Yes	24	26	
Vascular invasion			
No	29	14	<0.01**
Yes	11	42	
TNM stage			
I	19	10	0.003**
II	14	26	
III	7	20	
Tumor differentiation			
I–II	29	25	0.007**
III–IV	11	31	

*H2A.Z* H2A histone family member Z, *TNM* tumor, node, and metastasis, *AFP* alpha-fetoprotein.

**p < 0.01.

### Cell culture

In the current research, 4 HCC cell lines (Huh-7, MHCC-97H, SMMC-7721, and HepG2), HEK293T cell lines, and a normal human liver cell line (LO2) were purchased from the ATCC cell bank. All the cell lines were seeded in 6-well plates at a density of 1.5 × 10^5^ cells/well and maintained in Roswell Park Memorial Institute (RPMI)−1640 (A1049101, Gibco) supplemented with 10% fetal bovine serum (FBS). All the cells were cultured at 37 °C in a humidified atmosphere containing 5% CO_2_.

### Western blot analysis

In this study, total protein was extracted from cells and tissues subjected to sodium dodecyl sulfate-polyacrylamide gel electrophoresis (SDS-PAGE). Protein concentrations were transferred onto polyvinylidene difluoride (PVDF) membranes, and then membranes were blocked and incubated with primary antibody at 4 °C overnight. After washing three times with TBS-T solution for 10 min, the membranes underwent hybridization with a goat anti-rabbit IgG secondary antibody (dilution, 1:1000) at 37 °C for 1 h. After further washing, target protein levels were visualized using an enhanced chemiluminescence kit (No. 35055, Thermo Scientific). The antibody information is shown in Table S1.

### Transfection

For transfection, the cells were starved 2 h prior to transfection, using Lipofectamine 3000 (L3000015, Invitrogen) on the basis of the manufacturer’s protocols. Moreover, at 40 h after transfection, cells were treated with complete media for the indicated period before harvesting for further analysis. The transfection product information is shown in Table S2.

### Quantitative reverse transcription-polymerase chain reaction (qRT-PCR)

Total RNA was extracted using the RNeasy Kit (AM1924, Invitrogen) according to the manufacturer’s instructions, and reversely transcribed into cDNA using AMV reverse transcriptase, which was then used for amplification of the target genes. Furthermore, glyceraldehyde 3-phosphate dehydrogenase (GAPDH) was employed as an internal standard. Primer sequences used for qRT-PCR analysis are listed in Table S3.

### Cell migration and invasion assays

Cell migration and invasion assays were performed using Millicell Cell Culture Inserts (24-well plates; 8 µm pore size). Stably transduced cells were used for these assays. For the migration assay, cells in a serum-free medium were seeded on the upper chambers. For the invasion assays, the membranes of the upper chambers were coated with 8 µl Matrigel (No.A1413301, Gibco) in 32 µl RPMI-1640 medium for 3 h in a humidified incubator. The cells were then seeded in the coated upper chambers. RPMI-1640 medium containing 10% FBS was added to the lower chambers. Cells were incubated for 24 or 48 h for the migration assays, and 48 or 72 h for the invasion assays, respectively. Then, the cells on the lower membranes were stained using a Wright-Giemsa Stain kit (No.9990710, Thermo Scientific) and observed at ×100 magnification. Five fields were randomly selected and cells were counted upon observation under a light microscope, the number of cells of average per field was calculated finally.

### Apoptosis and cell cycle assay

Cells were trypsinized and centrifuged at 1500 rpm/min for 5 min. Cells were harvested and washed with Phosphate Buffered Saline (PBS) twice. Reagents for apoptosis detection were added, and then cells were incubated in dark for 30 min and subjected to flow cytometry analysis (FACS) (Beckman Coulter, Brea, CA, USA). In addition, cells were collected, washed with PBS, fixed with 75% ethanol at −20 °C overnight, and centrifuged at 1500 rpm/min for 5 min. Then, ethanol was removed and cells were washed with PBS twice. Propidium iodide (PI) and 500 μl of RNAse were added, and then cells were incubated in dark at 4 °C for 60 min. Lastly, cells were subjected to cell cycle analysis by FACS.

### Colony formation assay

The cultured cells at logarithmic growth were harvested and added to the six-well plates for the 14 or 21-day culture process at 37 °C in 5% CO2. After washing by PBS and fixing with 4% paraformaldehyde, cells were processed with crystal violet solution for staining, followed by manual counting.

### EdU assay

Cultured cells were placed at 5 × 10^4^ cells/well to the 96-well plates for treating with an EdU staining kit (RiboBio, Guangzhou, China). After that, cell nuclei were dual stained with DAPI solution, and then cells were subjected to final observation with a fluorescence microscope (Olympus, Tokyo, Japan).

Co-immunoprecipitation (Co-IP)

For the co-immunoprecipitation assay (Co-IP), the cells were lysed with modified TNE buffer (50 mM Tris [pH 8.0], 1% Nonidet P-40 [NP-40], 150 mM NaCl, 2 mM EDTA, 10 mM sodium fluoride, 10 mM sodium pyrophosphate) supplemented with 1 mg/l aprotinin, 1 mM sodium orthovanadate (Na_3_VO_4_) and 1 mg/l leupeptin. Lysates were centrifuged and cleared by incubation with 25 μl of Protein A/G gel for 1.5 h at 4 °C. The pre-cleared supernatant was subjected to IP using the indicated first antibodies at 4 °C overnight. Then, the protein complexes were collected by incubation with 30 μl of Protein A/G gel for 2 h at 4 °C. The protein complexes were resolved by SDS-PAGE. Subsequently, a western blot was performed. The antibody information is shown in Table S1.

### Chromatin immunoprecipitation(ChIP)

Cells were fixed with formaldehyde 1% for 15 min, then cells and nuclei were lysed. The recovered chromatin was sonicated: 10 cycles of 10 s (1 s on, 1 s off) and precleared. One hundred micrograms of chromatin were used for immunoprecipitation with 2 µg of antibodies of H2A.Z. Then, the chromatin was incubated with A/G beads for 2 h. Crosslinking was reversed by incubation of the beads with SDS at 70 °C and proteins were degraded with proteinase K. Finally, DNA was purified using the DNA purification kit (No.142095, CST), and ChIP was analyzed by qPCR using specific primers (Sangon Biotech, Shanghai). Primer sequences are shown in Table S3.

### RNA pull‑down assay

The synthesis of (RNA) was carried out with MAXIscript® Kit (Thermo Fisher Scientific, Inc.) according to the manufacturer’s instructions. The interaction between (RNA) and (protein) was examined using the Pierce™ Magnetic RNA-Protein Pull-Down Kit (Thermo Fisher Scientific, Inc.) following the instructions of the manufacturer. The RNA-binding protein complexes were washed, eluted, and could be analyzed by western blot analysis. The antibody information is shown in Table S1.

### RNA immunoprecipitation (RIP-seq and RIP-qPCR)

Collection of cells (cells selectively treated with formaldehyde, in vivo cross-linked protein-RNA complexes). Separate the nuclei and lyse the nuclei to precipitate. Chromatin fragmentation. The RNA binding protein of interest (RBP) and the binding RNA was immunoprecipitated together. Wash away unbonded substances. Purified RNA binding on RBP after immunoprecipitation. Reverse transcribe RNA into cDNA for analysis by qPCR or sequencing. The antibody information is shown in Table S1. The prime sequence information is shown in Table S3.

### Tumor xenograft experiments

A total of 18 BALB/c male nude mice (8 weeks old) were purchased from Shanghai X-B Animal. Ltd(Shanghai, China). All mice were kept in pathogen-free cages at 26–28 °C and at 50% humidity. H2A.Z expressing shH2A.Z, clone H2A.Z HepG2 cells, and control shRNA cells were resuspended in 100 µl PBS and subcutaneously injected into the right side of nude mice (3 × 10^6^ cells/mouse, 6 nude mice per group). Tumor volumes were measured after 7 days, and every 2 days afterward. Tumor volume was calculated using the formula: V (mm^3^) = width^2^ (mm^2^) × length (mm)/2. All the mice were sacrificed 3 weeks after the injection and tumors were harvested for analysis.

To generate a pulmonary metastasis mouse model, 3 × 10^6^ MHCC-97H cells expressing shH2A.Z, clone_H2A.Z, or control shRNA were resuspended in 100 μl PBS and injected into another set of BALB/c nude mice through the tail vein (6 nude mice per group). The lungs of the mice were collected after 7 weeks. Each lung was disposed into 30 sections, and H&E staining was performed to analyze the tumor clusters in the lung tissues (×100 magnification). All tumors were examined.

### In situ hybridization (ISH)

In situ hybridization was performed to detect the expression of lincZNF337-AS1 on formalin-fixed paraffin-embedded tissue sections with manufacturer’s procedures (servicebio, Hubei, China). The sections were dried at 65 °C for 3 h and then deparaffinized in xylene and ethanol at room temperature (RT) followed by a 10 min incubation with proteinase-k at 37 °C. After dehydration in ethanol, sections were hybridized with 40 nM double-DIG LNA™ lincZNF337-AS1 probes 55 °C for 1 h. After washing in SSC buffer at hybridization temperature and incubation with blocking solution for 15 min, the anti-DIG reagent sheep anti-DIG-AP (servicebio, Hubei, China) was applied and incubated for 60 min at RT. After washing in PBST, the sections were incubated with AP substrate NBT-BCIP (Roche) for 2 h at 30 °C and incubated in KTBT buffer to stop the reaction. Then sections were investigated and analyzed under microscopy.

### Immunohistochemistry

The sections were dried at 55 °C for 2 h and then deparaffinized in xylene and rehydrated using a series of graded alcohol washes. The tissue slides were then treated with 3% hydrogen peroxide in methanol for 15 min to quench endogenous peroxidase activity and antigen retrieval then performed by incubation in 0.01 M sodium citrate buffer (pH 6.0) and heating using a microwave oven. After a 1 h preincubation in 10% goat serum, the specimens were incubated with primary antibody overnight at 4 °C. The tissue slides were treated with a non-biotin horseradish peroxidase detection system according to the manufacturer’s instruction (servicebio, Hubei, China). Two different pathologists evaluated the immunohistological samples.

### Plasmid constructs

To generate potential eGFP fusion protein constructs with potential BCL6 protein domain, the sequences were amplified using RT-PCR and cloned into pcDNA3.1 plasmid. The DNA sequences of the exons of each truncated variant of lincZNF337-AS1 were synthesized (Genecreate, Hubei, China) and cloned into the pcDNA3.1 plasmid. Plasmids construct sequences are shown in Table S4.

### Transcriptome sequencing technology (RNA-seq)

Total RNA was extracted using the RNeasy Kit (AM1924, Invitrogen), and further treated with DNase to remove genomic DNA contamination. Isolation of mRNA was performed using the NEB. Next PolyA mRNA Magnetic Isolation Module (New England Biolabs, Ipswich, MA, USA) and the mRNA was then used for RNA-Seq library preparation with the NEB Next Ultra Directional RNA Library Prep Kit for Illumina (New England Biolabs, Ipswich, MA, USA). The library was then subjected to Illumina sequencing with paired-end 2 × 150 as the sequencing mode. Raw reads were filtered to obtain high-quality clean reads by removing sequencing adapters, short reads (length < 100 bp) and low-quality read using Cutadapt (v1.9.1) and Trimmomatic (v0.35). Then Fast QC is used to ensure high reads quality. The clean reads were mapped to the mouse genome (assembly GRCm38) using the HISAT2 software. Gene expression levels were estimated using FPKM (fragments per kilobase of exon per million fragments mapped) by String Tie. Ballgown, an R package, was used to measure differential gene expression. The false discovery rate (FDR) control method was used to calculate the adjusted *P*-values in multiple testing in order to evaluate the significance of the differences. Here, the only genes with an adjusted *P*-value < 0.05 were used for subsequent analysis.

### GSEA analysis

Global mRNA expression profiles of HCC samples from Gene Expression Omnibus (GEO) were subject to Gene Set Enrichment Analysis (GSEA) using GSEA 4.0.2 software available on (http:/www.broadinstitute.org/gsea/) to reveal the connection between H2A.Z expression and tumor-related characteristics pathway signature.

### Statistical analysis

In the present research, SPSS 19.0 software (IBM, Armonk, NU, USA), GraphPad Prism 6.0 software (GraphPad Software, La Jolla, CA), and R3.6.1 (AT&T Bell Laboratories, USA) were used to perform statistical analyses. Quantitative data were expressed as mean ± standard deviation (SD) and were examined by independent sample *t*-test. The counting data were analyzed by chi-square test. Multivariate Cox risk models were used to analyze risk factors for free survival and recurrence in patients with HCC. The Kaplan-Meier estimator was used to estimate the patients’ overall survival rates. *P* < 0.05 was considered statistically significant.

## Supplementary information

Table S1

Table S2

Table S3

Table S4

Table S5

Table S6

Table S7

Table S8

Table S9

Figure S1

Figure S2

Figure S3

Figure S4

Figure S5

Supplementary figures legends

## Data Availability

All data generated or analyzed during this study are included in this published article and its supplementary information files.
