# Supplementary material for: H2A.Z acetylation by lincZNF337-AS1 via KAT5 implicated in the transcriptional misregulation in cancer signaling pathway in hepatocellular carcinoma
Source: Cell Death Dis. 2021 Jun 12;12(6):609. doi: 10.1038/s41419-021-03895-2 (PMC8197763; doi:10.1038/s41419-021-03895-2)
Supplement: Supplementary file 1 — Table S1 [file 41419_2021_3895_MOESM1_ESM.docx]

**TableS1: Antibody information**

| REAGENT | SOURCE | IDENTIFIER |
| --- | --- | --- |
| Anti-BCL6 | Thermo Scientific | PA5-27390 |
| Anti-GFP | abcam | Ab290 |
| Anti-KAT5/TIP60 | Santa cruz | SC-166323 |
| Anti-TCF3 | Proteintech | 21242-1-AP |
| Anti-CDK14 | Proteintech | 21612-1-AP |
| Anti-IGF1 | Proteintech | 28530-1-AP |
| Anti-JUP | CST | CST#2309 |
| Anti-SPINT1 | Proteintech | 27593-1-AP |
| Anti-CDKN1A | abcam | Ab188224 |
| Anti-KAT3 | Santa cruz | SC-365219 |
| Anti-H2A.Z | abcam | Ab4174 |
| Anti-H2A.ZacK4+K7+K11 | abcam | Ab232908 |
| Anti-β-actin | Santa cruz | Sc-47778 |
| Anti-IgG | abcam | Ab172730 |
| HRP secondary antibody | abcam | Ab6721 |
| Anti-acetylation | abcam | Ab21623 |
