# Supplementary material for: H2A.Z acetylation by lincZNF337-AS1 via KAT5 implicated in the transcriptional misregulation in cancer signaling pathway in hepatocellular carcinoma
Source: Cell Death Dis. 2021 Jun 12;12(6):609. doi: 10.1038/s41419-021-03895-2 (PMC8197763; doi:10.1038/s41419-021-03895-2)
Supplement: Supplementary file 2 — Table S2 [file 41419_2021_3895_MOESM2_ESM.docx]

**TableS2:Transfection products information**

| REAGENT | SOURCE | IDENTIFIER OR SEQUENCE |
| --- | --- | --- |
| clone_H2A.Z-1 | ORIGENE | RC210691L2V |
| sh_NC | ORIGENE | TR30021 |
| sh_H2A.Z-1 | ORIGENE | TL312536V |
| clone_H2A.Z-2 | ORIGENE | [RC210691L4](https://www.origene.com/catalog/cdna-clones/lentiviral-particles/rc204775l2v/cltrn-nm_020665-human-tagged-orf-clone-lentiviral-particle)V |
| sh_H2A.Z-2 | Santa cruz | sc-62462-V |
| si_KAT5 | Santa cruz | sc-37966 |
| si_BCL6-1 | Santa cruz | sc-37966 |
| si_BCL6-2 | ORIGENE | SR300416 |
| si_lincZNF337-AS1-1 | Genecreate | GAGUGCAGUGGCAACAUCAUATT |
| si_lincZNF337-AS1-2 | Genecreate | GCCCUCCCAAAGUGAUGAGAUTT |
