# Supplementary material for: H2A.Z acetylation by lincZNF337-AS1 via KAT5 implicated in the transcriptional misregulation in cancer signaling pathway in hepatocellular carcinoma
Source: Cell Death Dis. 2021 Jun 12;12(6):609. doi: 10.1038/s41419-021-03895-2 (PMC8197763; doi:10.1038/s41419-021-03895-2)
Supplement: Supplementary file 3 — Table S3 [file 41419_2021_3895_MOESM3_ESM.docx]

TableS3 Primer sequences for qRT-PCR

| Variables | Primer sequences size |
| --- | --- |
| KAT5  KAT3  GAPDH  H2A.Z  LincZNF337-AS1  Exon1+2  Exon2+3  Exon3+4  Exon4+5  Exon full length  CDK14  CDKN1A  IGF1  JUP  SPINT1  TCF3 | 5’- GGGGAGATAATCGAGGGCTG -3’ 152bp  5’- TCCAGACGTTTGTTGAAGTCAAT -3’  5’- AGCCAAGCGGCCTAAACTC -3’ 144bp  5’- TCACCACCATTGGTTAGTCCC -3’  5’-GGAGCGAGATCCCTCCAAAAT-3’ 197bp  5’-GGCTGTTGTCATACTTCTCATGG-3’  5’- GGCGGTAAGGCTGGAAAGG -3’ 101bp  5’- TGTCGATGAATACGGCCCAC -3’  5’-CTACAGGCACTTTCCCTACATTTTC-3’ 291bp  5’- CTACAGGCACTTTCCCTACATTTTC -3’  5’- CGCGATCTGTCTCATTCCCT -3’ 109bp  5’- TGAGCTATGATGTTGCCACTG -3’  5’- GCTCAAGTGATCCTCCTGCT -3’ 200bp  5’- CTGGCAGGAGTAAAGCATGT -3’  5’- CAAAGGCCTGCATCATCACA -3’ 167bp  5’- CAGACAGAATCCAGAGGGCT -3  5’- CCTCTGGATTCTGTCTGCCT -3’ 189bp  5’- AGCTTCCAGAGTGACACAGG -3  5’- ATTCCCGCGATCTGTCTCAT -3’ 224bp  5’- CAGATGTGATGATGCAGGCC -3  5’- TGGGAAGTTGGTAGCTCTGAA -3 207bp  5’- CCAGGGTGCTTGTCCATGTA -3  5’- TGTCCGTCAGAACCCATGC -3 139bp  5’- AAAGTCGAAGTTCCATCGCTC -3  5’- GCTCTTCAGTTCGTGTGTGGA -3 133bp  5’- GCCTCCTTAGATCACAGCTCC -3  5’- TCGCCATCTTCAAGTCGGG -3 169bp  5’- AGGGGCACCATCTTTTGCAG -3  5’- AACTTGGCGCTAGTGGAGC -3 141bp  5’- TTCCCTCGTGAGGTAGTTGAT -3  5’- ACGAGCGTATGGGCTACCA -3 233bp  5’- GTTATTGCTTGAGTGATCCGGG -3 |
