# Supplementary material for: H2A.Z acetylation by lincZNF337-AS1 via KAT5 implicated in the transcriptional misregulation in cancer signaling pathway in hepatocellular carcinoma
Source: Cell Death Dis. 2021 Jun 12;12(6):609. doi: 10.1038/s41419-021-03895-2 (PMC8197763; doi:10.1038/s41419-021-03895-2)
Supplement: Supplementary file 4 — Table S4 [file 41419_2021_3895_MOESM4_ESM.docx]

**TableS4: Protein of bcl6 and lincZNF337-AS1 truncation sequences**

| Protein truncation of bcl6-1 | sequences |
| --- | --- |
| 15513_1 | CTATAGGGAGACCCAAGCTGGCTAGCATGTGCATCCAGTTCACCAGACACGCCAGCGAC |
| 15513_2 | ATGTCTCTGCTCCGCAGTCTGTTCAGGTTCAGCAGCACGTCGCTGGCGTGTCTGG |
| 15513_3 | CTGCGGAGCAGAGACATCCTGACCGACGTGGTCATCGTGGTGTCCAGAGAGCAGT |
| 15513_4 | GCCGCTGCAGGCCATCAGCACGGTCTTGTGGGCTCTGAACTGCTCTCTGGACACC |
| 15513_5 | TGATGGCCTGCAGCGGCCTGTTCTACAGCATCTTCACCGACCAGCTGAAGTGCAA |
| 15513_6 | CGGGGTTGATCTCGGGGTCCAGATTGATCACGCTCAGGTTGCACTTCAGCTGGTC |
| 15513_7 | CCCCGAGATCAACCCCGAGGGCTTCTGTATCCTGCTGGACTTCATGTACACCAGC |
| 15513_8 | ATCACGGCCATGATATTGCCCTCGCGCAGATTCAGGCGGCTGGTGTACATGAAGT |
| 15513_9 | AATATCATGGCCGTGATGGCCACAGCCATGTACCTGCAGATGGAACACGTGGTGGATAC |
| 15513_10 | TCCTCGCCCTTGCTCACCATGGTACCGATGAACTTCCGGCAGGTATCCACCACGTGTTC |
| Protein truncation of bcl6-2 | sequences |
| 15514_1 | CTATAGGGAGACCCAAGCTGGCTAGCATGAACCTCAAGACCCACACCAGAATCCA |
| 15514_2 | CTCGCACTTGTAGGGCTTCTCGCCGCTGTGGATTCTGGTGTGGGTCTTGAGGTTC |
| 15514_3 | GCGAGAAGCCCTACAAGTGCGAGACATGCGGCGCCAGATTCGTGCAGCATCTGAG |
| 15514_4 | CTTCTCCCCTGTGTGGATCAGCACGTGGGCTCTCAGATGCTGCACGAATCTGGCG |
| 15514_5 | GTGCTGATCCACACAGGGGAGAAGCCTTATCCTTGCGAGATCTGCGGCACCCGGT |
| 15514_6 | TGTGCACGGTCTTGTGGCTGGCCAGATTGTGTCTGAACCGGGTGCCGCAGATCTC |
| 15514_7 | GCCACAAGACCGTGCACACCGGCGAAAAGCCTTACCGGTGCAATATCTGTGGCGC |
| 15514_8 | TATGAATTCTCAGGTGGGACTTCAGCCGGTTGAACTGGGCGCCACAGATATTGCA |
| 15514_9 | CCACCTGAGAATTCATACCGGCGAGAAACCATATCACTGCGAGAAGTGCAACCTG |
| 15514_10 | TCCTCGCCCTTGCTCACCATGGTACCATGCCGGAAGTGCAGGTTGCACTTCTCGC |
| Protein truncation of bcl6-3 | sequences |
| 15515_1 | CTATAGGGAGACCCAAGCTGGCTAGCATGGCCCCTGGCTGTGAAAGCAGAGCCTTTGCT |
| 15515_2 | CTGGCAGGAGGTGTAGACAGGCCGCTGTACAGGCTAGGAGCAAAGGCTCTGCTTT |
| 15515_3 | TCTACACCTCCTGCCAGCTACAGCATGTACAGCCATCTGCCTGTGTCCAGCCTGC |
| 15515_4 | AGGCATCCGCACGTCCCTGAATTCCTCGTCGCTGAACAGCAGGCTGGACACAGGC |
| 15515_5 | GGGACGTGCGGATGCCTGTGGCCAATCCATTTCCTAAAGAGCGGGCCCTGCCTTG |
| 15515_6 | GTCTGCTGTACTCGCCAGGAACAGGTCTAGCGCTATCGCAAGGCAGGGCCCGCTC |
| 15515_7 | TGGCGAGTACAGCAGACCCACACTGGAAGTGTCCCCAAACGTGTGCCACAGCAAC |
| 15515_8 | CTGGCCTCTTCGGGGATTGTCTCTTTGGGGCTGTAGATGTTGCTGTGGCACACGT |
| 15515_9 | ATCCCCGAAGAGGCCAGATCCGACATGCACTACTCTGTGGCCGAGGGCCTGAAAC |
| 15515_10 | GGGGAAGTAAGGGGCGTTTCTAGCAGATGGAGCAGCAGGTTTCAGGCCCTCGGCC |
| 15515_11 | ACGCCCCTTACTTCCCCTGCGACAAGGCCAGCAAAGAGGAAGAGAGGCCCAGCAGCGA |
| 15515_12 | AGGGGAGCGTTTGGAGGCTCGAAGTGCAGGGCGATCTCATCCTCGCTGCTGGGCCTCTC |
| 15515_13 | CCTCCAAACGCTCCCCTGAACAGAAAAGGCCTGGTGTCCCCTCAGAGCCCTCAGAAGTC |
| 15515_14 | TTGCTGCTGCAGCTCTCGGTAGGGCTGTTAGGCTGGCAGTCAGACTTCTGAGGGCTCTG |
| 15515_15 | GAGAGCTGCAGCAGCAAGAACGCCTGTATCCTGCAAGCCTCTGGCAGCCCTCCAGCCAA |
| 15515_16 | TTGTACTTTTTCCAGTTGCAGGCCTTGGGATCTGTAGGAGACTTGGCTGGAGGGCTGCC |
| 15515_17 | AACTGGAAAAAGTACAAGTTCATCGTGCTGAACTCCCTGAACCAGAACGCCAAGCCTGA |
| 15515_18 | GCTCTGGGGCTCAGTCTGCCCAGTTCAGCCTGTTCAGGTCCCTCAGGCTTGGCGTTCTG |
| 15515_19 | AGACTGAGCCCCAGAGCCTATACAGCTCCTCCTGCTTGCCAGCCTCCTATGGAACCCGA |
| 15515_20 | TCCTCGCCCTTGCTCACCATGGTACCCTGCAGATCCAGGTTCTCGGGTTCCATAGGAGG |
| Exon1+2 | sequences |
| 15626_1 | CTATAGGGAGACCCAAGCTGGCTAGCCGGCGCGGGTGGAATTCCCGCGATCTGTCTC |
| 15626_2 | TCTCGCGGGATCTCACTTTTCTCGGCGGCGCAGGGAATGAGACAGATCGCGGGAA |
| 15626_3 | AGTGAGATCCCGCGAGACTCCCAGGGGTCTCATTCTGTCACCCAGGTTGGAGTGC |
| 15626_4 | AGGGGTTCAAGGCTGCAGTGAGCTATGATGTTGCCACTGCACTCCAACCTGGGTG |
| 15626_5 | TGCAGCCTTGAACCCCTGGGCTCAAGTGATCCTCCTGCTTCAGCCTCCTGAGTAG |
| 15626_6 | TTCCACCACACTGGACTAGTGGATCCCTGTAGCCCCAGCTACTCAGGAGGCTGAA |
| Exon2+3 | sequences |
| 15627_1 | CTATAGGGAGACCCAAGCTGGCTAGCGGTCTCATTCTGTCACCCAGGTTGGAGTGCAGT |
| 15627_2 | CCCAGGGGTTCAAGGCTGCAGTGAGCTATGATGTTGCCACTGCACTCCAACCTGG |
| 15627_3 | AGCCTTGAACCCCTGGGCTCAAGTGATCCTCCTGCTTCAGCCTCCTGAGTAGCTG |
| 15627_4 | GCAGGCCTTTGAAAATGTAGGGAAAGTGCCTGTAGCCCCAGCTACTCAGGAGGCT |
| 15627_5 | CATTTTCAAAGGCCTGCATCATCACATCTGCCACAGCATTCTCTATCATTGAGGC |
| 15627_6 | AGCTGCAAAATATTTCACAGGTGATTTCCTGAGAAAATGCCTCAATGATAGAGAA |
| 15627_7 | TGAAATATTTTGCAGCTAACCACCTACCTGTGCCCCACCAGCCAACATGCTTTAC |
| 15627_8 | TTCCACCACACTGGACTAGTGGATCCCTGCCTGGCAGGAGTAAAGCATGTTGGCTG |
| Exon3+4 | sequences |
| 15628_1 | CTATAGGGAGACCCAAGCTGGCTAGCGCACTTTCCCTACATTTTCAAAGGCCTGC |
| 15628_2 | TGATAGAGAATGCTGTGGCAGATGTGATGATGCAGGCCTTTGAAAATGTAGGGAA |
| 15628_3 | CACAGCATTCTCTATCATTGAGGCATTTTCTCAGGAAATCACCTGTGAAATATTT |
| 15628_4 | ATGTTGGCTGGTGGGGCACAGGTAGGTGGTTAGCTGCAAAATATTTCACAGGTGA |
| 15628_5 | GCCCCACCAGCCAACATGCTTTACTCCTGCCAGGCAGGTTCTCTGTAGCCCTCTG |
| 15628_6 | AACTTGTTCGTGAAGATATAACAGAGGCAGACAGAATCCAGAGGGCTACAGAGAA |
| 15628_7 | ATCTTCACGAACAAGTTATGCAGCCTCAACTAAAGCTTGTAACAAAGCAAAGTTA |
| 15628_8 | TATATCTCACCATCCATCAGAGGACACACTCAGGAGAGTAACTTTGCTTTGTTAC |
| 15628_9 | ATGGATGGTGAGATATAACTTCAAGATGAAGCCTCACCCACACTCCCTGTACAAA |
| 15628_10 | TTCCACCACACTGGACTAGTGGATCCGTGAGAAGCGTTTTTGTACAGGGAGTGTG |
| Exon4+5 | sequences |
| 15629_1 | CTATAGGGAGACCCAAGCTGGCTAGCGTTCTCTGTAGCCCTCTGGATTCTGTCTGCCTC |
| 15629_2 | TTTAGTTGAGGCTGCATAACTTGTTCGTGAAGATATAACAGAGGCAGACAGAATCCA |
| 15629_3 | ATGCAGCCTCAACTAAAGCTTGTAACAAAGCAAAGTTACTCTCCTGAGTGTGTCCTCTG |
| 15629_4 | TGTGGGTGAGGCTTCATCTTGAAGTTATATCTCACCATCCATCAGAGGACACACTCAGG |
| 15629_5 | ATGAAGCCTCACCCACACTCCCTGTACAAAAACGCTTCTCACCTGTGTCACTCTGGAAG |
| 15629_6 | TGCACATCAGAGGCAACAGCAACTACAATTTTCTGATCAAAGCTTCCAGAGTGACACAG |
| 15629_7 | GTTGCCTCTGATGTGCAAGTCCAAGATTCTTGGGCCGCAGGACATGTTCTGCATATATT |
| 15629_8 | TTCCACCACACTGGACTAGTGGATCCTGGTCTATTCTCCAGGAATATATGCAGAACATG |
| Exon full length | sequences |
| 15630_1 | CTATAGGGAGACCCAAGCTGGCTAGCCGGCGCGGGTGGAATTCCCGCGATCTGTCT |
| 15630_2 | CTCGCGGGATCTCACTTTTCTCGGCGGCGCAGGGAATGAGACAGATCGCGGGAAT |
| 15630_3 | AAGTGAGATCCCGCGAGACTCCCAGGGGTCTCATTCTGTCACCCAGGTTGGAGTG |
| 15630_4 | GGGGTTCAAGGCTGCAGTGAGCTATGATGTTGCCACTGCACTCCAACCTGGGTGA |
| 15630_5 | CTGCAGCCTTGAACCCCTGGGCTCAAGTGATCCTCCTGCTTCAGCCTCCTGAGTA |
| 15630_6 | GCCTTTGAAAATGTAGGGAAAGTGCCTGTAGCCCCAGCTACTCAGGAGGCTGAAG |
| 15630_7 | CCTACATTTTCAAAGGCCTGCATCATCACATCTGCCACAGCATTCTCTATCATTG |
| 15630_8 | GCAAAATATTTCACAGGTGATTTCCTGAGAAAATGCCTCAATGATAGAGAATGCT |
| 15630_9 | CCTGTGAAATATTTTGCAGCTAACCACCTACCTGTGCCCCACCAGCCAACATGCT |
| 15630_10 | ATCCAGAGGGCTACAGAGAACCTGCCTGGCAGGAGTAAAGCATGTTGGCTGGTGG |
| 15630_11 | TCTGTAGCCCTCTGGATTCTGTCTGCCTCTGTTATATCTTCACGAACAAGTTATG |
| 15630_12 | GAGTAACTTTGCTTTGTTACAAGCTTTAGTTGAGGCTGCATAACTTGTTCGTGAA |
| 15630_13 | ACAAAGCAAAGTTACTCTCCTGAGTGTGTCCTCTGATGGATGGTGAGATATAACT |
| 15630_14 | GTTTTTGTACAGGGAGTGTGGGTGAGGCTTCATCTTGAAGTTATATCTCACCATC |
| 15630_15 | ACTCCCTGTACAAAAACGCTTCTCACCTGTGTCACTCTGGAAGCTTTGATCAGAA |
| 15630_16 | CTTGGACTTGCACATCAGAGGCAACAGCAACTACAATTTTCTGATCAAAGCTTCC |
| 15630_17 | TGATGTGCAAGTCCAAGATTCTTGGGCCGCAGGACATGTTCTGCATATATTCCTG |
| 15630_18 | TTCCACCACACTGGACTAGTGGATCCTGGTCTATTCTCCAGGAATATATGCAGAA |
