# Supplementary material for: H2A.Z acetylation by lincZNF337-AS1 via KAT5 implicated in the transcriptional misregulation in cancer signaling pathway in hepatocellular carcinoma
Source: Cell Death Dis. 2021 Jun 12;12(6):609. doi: 10.1038/s41419-021-03895-2 (PMC8197763; doi:10.1038/s41419-021-03895-2)
Supplement: Supplementary file 5 — Table S5 [file 41419_2021_3895_MOESM5_ESM.docx]

tableS5: PCR primers for in vitro amplification

| truncation | | sequences |
| --- | --- | --- |
| 1+2（207 bp） | Sense | F：gctagccggcgcgggtggaattccc  R：ggatccctgtagccccagctactcagg |
|  | Antisense | F：ggatccctgtagccccagctactca  R：gctagccggcgcgggtggaattcccgc |
| 2+3（286bp） | Sense | F：gctagcggtctcattctgtcacccag  R：ggatccctgcctggcaggagtaaagc |
|  | Antisense | F：ggatccctgcctggcaggagtaaagc  R：gctagcggtctcattctgtcacccag |
| 3+4（350 bp） | Sense | F：gctagcgcactttccctacattttca  R：ggatccgtgagaagcgtttttgtaca |
|  | Antisense | F：ggatccgtgagaagcgtttttgtaca  R：gctagcgcactttccctacattttca |
| 4+5（311bp） | Sense | F：gctagcgttctctgtagccctctgg  R：ggatcctggtctattctccaggaat |
|  | Antisense | F：ggatcctggtctattctccaggaat  R：gctagcgttctctgtagccctctgg |
| Full length（662 bp） | Sense | F：gctagccggcgcgggtggaattcccg  R：ggatcctggtctattctccaggaat |
|  | Antisense | F：ggatcctggtctattctccaggaat  R：gctagccggcgcgggtggaattcccg |
