# Supplementary material for: H2A.Z acetylation by lincZNF337-AS1 via KAT5 implicated in the transcriptional misregulation in cancer signaling pathway in hepatocellular carcinoma
Source: Cell Death Dis. 2021 Jun 12;12(6):609. doi: 10.1038/s41419-021-03895-2 (PMC8197763; doi:10.1038/s41419-021-03895-2)
Supplement: Supplementary file 6 — Table S6 [file 41419_2021_3895_MOESM6_ESM.docx]

TableS6 Primer sequences for chip-qPCR of H2A.Z

| Variables | Primer sequences size |
| --- | --- |
| CDK14  CDKN1A  IGF1  JUP  SPINT1  TCF3 | 5’- GCTCACTAGCCGGTGAAAGT -3 212bp  5’- CCATTTTCAGCCTGGACTTC -3  5’- AGGAAGGGGATGGTAGGAGA -3 150bp  5’- CTCCCAGCACACACTCACAC -3  5’- TGATTCCCCAGGAAGATTTG -3 216bp  5’- TTGCTCACACAACCACAACA -3  5’- CCAAGAGGGCAGAACTGAAG -3 177bp  5’- GAGCCCAGAAGGAGGAAAAT -3  5’- GCCCTGGAAAGCTCCTACTT -3 173bp  5’- GCCCTGAGGAGTCTTGTCTG -3  5’- GGCTACGTCAGACCCATTTT -3 218bp  5’- AGGCGGGGTCACTTTTTACT -3 |
