# Supplementary material for: H2A.Z acetylation by lincZNF337-AS1 via KAT5 implicated in the transcriptional misregulation in cancer signaling pathway in hepatocellular carcinoma
Source: Cell Death Dis. 2021 Jun 12;12(6):609. doi: 10.1038/s41419-021-03895-2 (PMC8197763; doi:10.1038/s41419-021-03895-2)
Supplement: Supplementary file 7 — Table S7 [file 41419_2021_3895_MOESM7_ESM.docx]

TableS7 Primer sequences for chip-qPCR of bcl6

| Variables | Primer sequences size |
| --- | --- |
| CDK14  CDKN1A  IGF1  JUP  SPINT1  TCF3 | 5’-GCAACTCTGGGCTGAGAACT -3 170bp  5’-GCAACTCTGGGCTGAGAACT -3  5’- CACCTTTCACCATTCCCCTA -3 236bp  5’- GCAGCCCAAGGACAAAATAG -3  5’- TATGGCAGCCCTAGCAAACT -3 177bp  5’- CATTGGAGGCTGCATTTCTT -3  5’- CGTCAGGAACCCCTCTCTCT -3 197bp  5’- CTCGTAGTAGGCCCTCATGG -3  5’- GAAGACTGCCAGATCCGAGA -3 173bp  5’- CAGCTGACATCCCTTCCCTA -3  5’- AATCCCGTGAGGTCCAGAG -3 177bp  5’- CCAGACTGCTCTGCCTTAGC -3 |
