# Supplementary material for: H2A.Z acetylation by lincZNF337-AS1 via KAT5 implicated in the transcriptional misregulation in cancer signaling pathway in hepatocellular carcinoma
Source: Cell Death Dis. 2021 Jun 12;12(6):609. doi: 10.1038/s41419-021-03895-2 (PMC8197763; doi:10.1038/s41419-021-03895-2)
Supplement: Supplementary file 8 — Table S8 [file 41419_2021_3895_MOESM8_ESM.docx]

# Homer *de novo* Motif Results (HepG2_H2AZ/)

| Rank | Motif | Best Match | P-value | log P-pvalue | % of Targets | % of Background |
| --- | --- | --- | --- | --- | --- | --- |
| 1 | 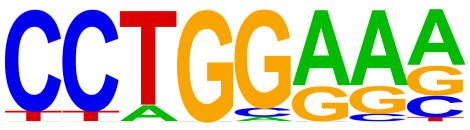 | Bcl6 | 1e-52 | -1.210e+02 | 35.70% | 13.60% |
| 2 | 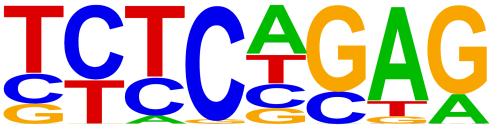 | CEP3 | 1e-32 | -7.410e+01 | 53.70% | 33.13% |
| 3 | 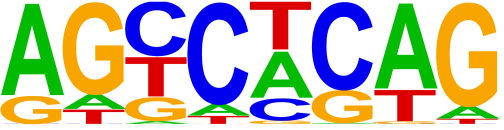 | MET28 | 1e-31 | -7.314e+01 | 54.30% | 33.84% |
| 4 | 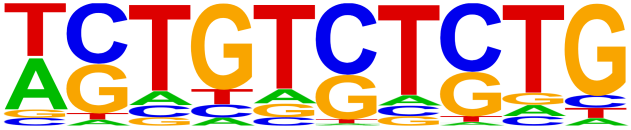 | CA-repeat | 1e-24 | -5.595e+01 | 29.90% | 15.25% |
| 5 | 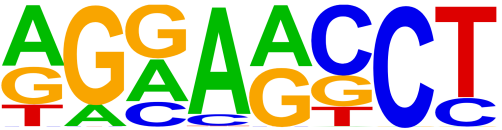 | che-1 | 1e-21 | -4.974e+01 | 26.00% | 13.00% |
| 6 | 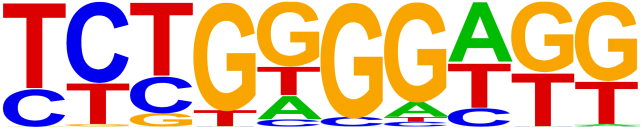 | ESRP2 | 1e-21 | -4.938e+01 | 12.50% | 3.91% |
| 7 | 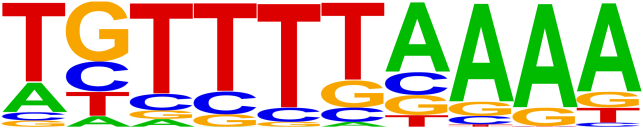 | Unknown6 | 1e-20 | -4.811e+01 | 39.20% | 23.93% |
| 8 | 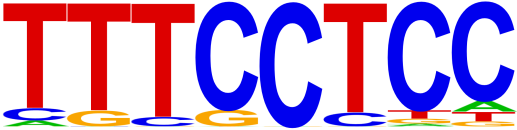 | PF10_0068 | 1e-16 | -3.750e+01 | 21.60% | 11.13% |
| 9 | 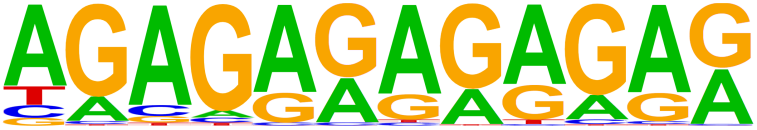 | GA-repeat | 1e-15 | -3.573e+01 | 62.60% | 48.38% |
| 10 | 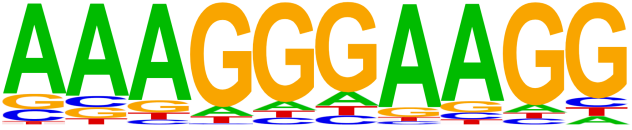 | PCBP2 | 1e-15 | -3.520e+01 | 13.00% | 5.29% |
