# Supplementary material for: H2A.Z acetylation by lincZNF337-AS1 via KAT5 implicated in the transcriptional misregulation in cancer signaling pathway in hepatocellular carcinoma
Source: Cell Death Dis. 2021 Jun 12;12(6):609. doi: 10.1038/s41419-021-03895-2 (PMC8197763; doi:10.1038/s41419-021-03895-2)
Supplement: Supplementary file 9 — Table S9 [file 41419_2021_3895_MOESM9_ESM.docx]

**List of promoter sites and sequences of H2A.Z and BCL6 binding downstream target genes analyzed by chip-seq**

**>HepG2_BCL6_Peaks_17310:IGF1:12:102416997-102417635**

GGAGAAGAAGGAGGCCACCTACAAGCCGAGGGGAGGAGCCCATGACAGAGCCTTCTCTAAGGCCCTTGCAAGGGCTGATACTTTGATTTTGGACTTTCAACTCCCAGAACTATGAAAAAATCAATTTCTGTGGTTTAAACTACCTAGCCTATGATACTTTGTTATGGCAGCCCTAGCAAACTTATACATCTTTTATATCTGATTCCAAAGTGCAAAAAAGATGAAGAGTGCAGTGACCTCTTCTCAAAAGTTACCTCACAGACCTCACAGGGTTTTAGGCAATACCGGATGCCCTTTGGCCTAAACCCTGGACTTGACTAAGAAATGCAGCCTCCAATGACATTGCGGGAAAAGGGAATCTGGGAACTTCTATGACACAATTCAGTCTTGCTGAGCATTTGGGGCTAATATTTAACTCTGAACATATATTGACATAGGCAATTCTTCCATAACAGATTCATACAAAATTTAAAAATGCATATAGAAGCCTTAATTTTTATTTAAATTCTTTTATTTAATTGTGTTTTAGAGGCAGAGAATAGTGTGTCTTTTTTTGCCTCTTTTATAATTTTTATTTTTTTTTTTCATTTTTGCCACTGTCTTTCTTTGCGCTTTCTAGGGCATTACATTTTTCTTTT

**>HepG2_BCL6_Peaks_22911:SPINT1:15:40845307-40846582**

GACCCGGCTAATTTTTTTTTTTTTTTTTTTTTTTTTTTTTTTGGTACAGACGGGGTTTCACTATGTTGGCCAGGCTGGTCTTGAACTTCTGAACTCAGGTGATCCACCCACCTTGGCCTCCCAAAGTGCTGGGATTATTTCTACCTGAAGCCCTCCTTAGAAGTAGGCAGTACAGCCGAATCAAGCCTGGTGATCAATGGGGTGACAGATGTCACAGCCAGATCGCCTTCACATCGGTAGTAGGCAGTACAAACAGTAAACAAATGAAAGGGGACATGAGTAGGGAGGGCAGGGCCCAGGCTCTGGCTCCTGAACTCAGGATGTTCAGAGGTGATGGGCTGGTGCTCACAGGGAGGCCTTTGGCTCTGGGCTGTGTGTACTGGAGGGGAGAGGAAGTCCCCAGGACTCCTGACCTCTGGCACTGATAGGCTGATTGAAGGAGTGAGCTGTCTGGATGGTCTGGTGACTAGAAAGGCACCTTGGCCAAGCACTGGTCTGGGCACACCGATGCCCTGTCAGGTGAGCCTGGCCATAGTCTCCTCCACTGGCTGGACTTGGGACTGCTTAGCATCTCTCTACCCCCTAGGAATGCATTGGCCTTTTGGTGGTCCAGGAAGACTGCCAGATCCGAGACTAGAAAACAAGCCCAGTCTGCGGTTGATCCAAAGGGAGAGAAGTGATGTTGTAGGCACAGAAGCCTAGAGGCTCTGGGGTGGGGGATGAGGAGGGTTGTGGGGTGAGTCAGCCTGTTGGTATGTATACCTAGTAGGGAAGGGATGTCAGCTGTCCAGGCAGGTGTGTGTGCATGCGCATGAGCCAGACAGGGCTCTCCCTGCTTAGAATCTGGTAGCCAAACCTTGTTTACTTCCATAGATAAAGGCAAAGCGGAGGCCAGGGCTCCTCCTCTTTCTTCCCCGGACTGAACTGTCTGCTGGGATCAGGGCCAGTCCAGAGCCTTGGTTCCCCCTTTCTCTCTACCTGCTGTTCTGGCCAGGTGGGGCTCCAGGGCAGGGGTAGGTCACCTCTCATAACCTCTGTGAGCACTTTACAGCTTACTGATTGCTGTGCCACACATCCTCTCACTCAGGCCTCATCACAACAACTTCCAAAAAGGGTGCTACTAGCAGTCATCCCACTTTTTAGATTAGGAAACTAAGACTCCCATGTTAAAGAATTTGCCCAGGGTAACACTGCCCATAAATGGTAGGCCTGAGATTCACCCCAGGGGAGACCTCCAAAAAAAGTCCTGTGCTGTTTTTCACACAAGACCATCTC

**>HepG2_BCL6_Peaks_30011:JUP:17:41772253-41774282**

TCTGAGGTCCCCTGGGCCCACCCTCAACTACTGGGGCTCCCTGGGAAAGGCTGCAGGGAGGAGGCACCCAAGCCATTGGGCAGGAAACTGGGCGGGGCAGGGAAGCGGTCTGACAGGTTTCTCTGGAGCGCCCCAGGCTCCCTCAGAGACTCCAGGATCTGGCTTTCCTACAGGTTGGGCAGGATCACACATGGCCCTGAGGTCCAGACCCTAAGGCCCCCTCTCTCCCAGCCCTCCCAGTCCCTAGACGCCTCTTTGAGTCCCACAGAGCAATTCAAATCTCAGCTACTCACTACTGGGGCAGTTCCTCTCCCCACAGAACCTCAGTGTCCTCATCTGTAAAGCGGGGTCACGCCACCTCCCTCAGAGGTGGTTGCGAGGACCTGGTGCATAGCAGGTGCTCAGAGGCCACGGCCATTATCATCAGCACTGCCTCTCCTCCAGAAGTGCTTCCTCACCTCCTGGTCTCCTCGAGCCCAGGCTAGGGATCTGGGGTGGGGCAGGCCTGTGGGTGGTCACTGCCGTCAGGAACCCCTCTCTCTCTCTGCAAAGGTTTAGGCGCACCTGAAGGTTAATGAGTAGCAGGGCCGAACTTTGTACCAGGGAGGTGAACTTCCCGCTTGGTCTGTGTTTGGCTCCACTGTCCTGGGAACTGACCTGCAGAGGTCAGAGGCTGCGGGGAGAATGGACGCCGTGATCCCATGAGGGCCTACTACGAGCCAGACCTTTCACAGACACCGTCATGCTCATGGGCGGGTTTCAAAGCCCATGCACTGGTCCCCCGCGTACAGATGCAGAAACTGAGGTTAGAAATGGCAGAGCTGGGACTGGGACCAGGTGTGAGGGCACCAAGGTCTGTAGTCTTTGCACATCCCGGCGCAGCCTCAGTGAGTAAGCAGAACTCAGGCTGGTCCACCCATGAAGCCAGGCAGCACCCGGGTTGTCTCACTGTGATGGGGACCAGGTGAGGGTGGGGTGGCTGGGGCAACCTGGCCCCACCCCAGGAACAAAGATCACCAAGGATAAGTCTTCCCTGTGACTGCCCCAGTGCCGCCCTGGCCCTCCAGGAGGATGACGAATGGTCCTGCAGGTCTCCCAGCCTTTTATGGCAGGCAGCCGTGAGCACACCCGGAACAGGCAGAGGCAGGGGCCAGGCCAGCTCCCAGCTGCCTCCTTCCTCTCCTCCCGCAATCCCTAGTGACTCAGAGTTTCAGGCTGATTAAACAGAAGCAGTCCCGCCACCTCAGACGGCAGACCCCAGACGACAGGCTCGGGCCGCCAGATGTGCACAGCTGCTTCCCAGGCATTCGAAAGTGTAGCTATTTCAGAAGGAGCCCCCCCAACCAGCCCCCTTTCCACACGCACCCCTTCCCCACACCCCTTCCCCAGCAACAGAGGGGAATGGCCACTGGCAGGAAACAGGGGCAATGGTGGGGGGCGGGGGGCGGGGTGCTCGGAGAAGGCAGACATGGGAAAACCCTACTAAAATCCTGAGCTCCTCGTGCTGTGCCGCCTTCCCCAACCATTTCCCTGCCCCAAGGGCAAGTCCCAAGAGAGAGCAGAGGAGAGTTTGGAAGAGAAGCTGCCCCCAGGAGAGAAGGAAGGTGCAAGTGTACAAGTAAACACGGTAGCAATAACCCACTGAATGCCGCTCTGCTGGGCTCAAGGCTGAACGACATCTGGACACTGCTGGACATCTGCAGCTCTGGTCAACAAACACACTGCATCCCAGCCAGAGGGCCCTCCTGCATAGACAGTGCCTAACCCTGGGGCTTCTCAGCTAAGGGAGAGGGAAGCGGGCCTCACTCCAAACAAGGGTCACCCCTTGCCGGCCTCACATCTAAAGGGACCACCACAGTCAAGCTGAGGAACTTCCTCAGCAGGCCCCTCACCACCCCCACCAGCCCAGGTCAACCGCCAGGAGACTGCTGAGGGCTAGACAGCTACCCAGGGAGAGACAGAAGCCACAGGATGCCATGGGGGGGTGGGGGGGTGGACGCCCAGTGGCCTTTATTATTATGTGAATCTC

**>HepG2_BCL6_Peaks_34198:TCF3:19:1652509-1653554**

AGCGGCGTGCGCGGTGCCCGCGGTGCCCGCCGCCGCGTCGGCTCCGGCCCGCTACGCCCGCAGCCGCCGCCGCTGCCTCATCTTCCTGCGGCGGGAGACATGTTCCGCCCCCCGCCCGCGCCGCCCCGCCCCGCCCCGTGCAGGCCCCGCCCCTGCCCCGCCCCCGAGTGCCCCGCCCGGCGGCCCACGCGGATCCCTCCGCCACCTCCGGGCGGCTCGGGCCCGAACGCCCTAGCTCGGCCTCTCGAGCACCCTCGTGAGGACCCCGAATCCCGTGAGGTCCAGAGCCTGGGAGCGGGGACGCGCAGAGGAGGCGGTCGGGCCACGGCGCGGGGAGAAATCACGGACTATCCCCGTCCGCGGAAGCACACACGAGCTGTGCGCTTAGTCCATGACGCAAGCGAGTAAGGCCCGTGAGATTGAAAGCTAAGGCAGAGCAGTCTGGTCAATCGGAAGCCGCGAAAGTCTGATGGGCGGGGATCCTAGCCATTCGTGGTGAGGCCCCGCCTCCTTTCTTCTCGGCCCCGCCCCTCAGCAGAGGCGGGACTCTGCGAGCGAGAGGCCGCGAGAGGCGGCCGGGGTGGGTCCTGGAGTTTTGTTCTCAGGTTGGCGTGGCCGCCCGCGCGGAGCCTTCTGCTTGGTTATATTTGCGTTCCTCGGGCCGGCCCCGCGTGCTGAGTGGTGCGAGCGGGTATCACGGCCCCGAGGGGGCTACGTCAGACCCATTTTCCCGGCGGGAAAACCGACTCTGGCTCCGGCTCTGGGTCAGAGAGCCCCGCGGGGAGTCTCAGCGGTGCCTTTGCCGACTTGAGTCTCCGTCTCGGCATCTGTGAATTTGGACCTGACTTTGGACAGAACTCAGGCCAGATCTGGCTTCCTGATTCTTAGTAGTCCGTGTCTACATTTTAGTAAAAAGTGACCCCGCCTAGGATCGGGCGCCGTGGCTCCTGCCTGTGATCCCAGCGCTTTGAAGGAGGCCGAGGCGGGAAGATCGCTTGAGGCCAGGAGTTCGAGAACAGCCTGAACAACATAGTGAAACCCCCCC

**>HepG2_BCL6_Peaks_61369:CDKN1A:6:36675539-36681307**

GCGATTCTCCCACCTCAGCCACCTGAATACCTGGGACTACAGGTGCCCACCACCATGCCCGGCTGATTTTTGTATTTTTAATGGAGACGGGGTTTCACCATATTGGCCAGGCTGGTCTCAAAACTCCTGACCCTGTGATCTGCCCGCCTCGGCCTCCCAAAGTGCTGGGATTACAGGCGTAAGCCACCACGCCCGGCCAGTATATATTTTTAATTGAGAAGCAAAATTGTACTTCAGATTTGTGATGCTAGGAACATGAGCAAACTGAAAATTACTAACCACTTGTCAGAAACAATAAATCCAACTTTTTGTGCAAAAAAAAAAATACAAATATTAGCTGGGCATGGTGGTGCATGCCTGTAATCCCAGCTACTCGGGAGGCTGAGGCAGAATTGCTTGAACCTGGGAGGCGGAGACTGCAGTGAGCTGAGATTGTGCCACTGCTGACTTTGTCTCAAAAAACAAAACAAAACAAAAAAACAAAATGAAAACAAAAAGCCAGGGCTGCCTCTGCTCAATAATGTTCTATCTTTGTTCCGCCTCTTCTCTGGGGTCTCACTTCTTGGGAGCCTGTGTGAAGGTGAATTCCTCTGAAAGCTGACTGCCCCTATTTGGGACTCCCCAGTCTCTTTCTGAGAAATGGTGACATTGTTCCCAGCACTTCCTCTCCCTTCCTAGGCAGCTTCTGCAGCCACCACTGAGCCTTCCTCACATCCTCCTTCTTCAGGCTTGGGCTTTCCACCTTTCACCATTCCCCTACCCCATGCTGCTCCACCGCACTCTGGGGAGGGGGCTGGACTGGGCACTCTTGTCCCCCAGGCTGAGCCTCCCTCCATCCCTATGCTGCCTGCTTCCCAGGAACATGCTTGGGCAGCAGGCTGTGGCTCTGATTGGCTTTCTGGCCGTCAGGAACATGTCCCAACATGTTGAGCTCTGGCATAGAAGAGGCTGGTGGCTATTTTGTCCTTGGGCTGCCTGTTTTCAGGTGAGGAAGGGGATGGTAGGAGACAGGAGACCTCTAAAGACCCCAGGTAAACCTTAGCCTGTTACTCTGAACAGGGTATGTGATCTGCCAGCAGATCCTTGCGACAGGGCTGGGATCTGATGCATGTGTGCTTGTGTGAGTGTGTGCTGGGAGTCAGATTCTGTGTGTGACTTTTAACAGCCTGCTCCCTTGCCTTTTTCAGGGCAGAAGTCCTCCCTTAGAGTGTGTCTGGGTACACATTCAAGTGCATGGTTGCAAACTTTTTTTTTTAAAGCACTGAATAGTACTAGACACTTAGTAGGTACTTAAGAAATATTGAATGTCGTGGTGGTGGTGAGCTAGAAGTTATAAAAAAAATTCTTTCCCAAAAACAACAACAAAAAGAATTATTTCATTGTGAAGCTCAGTACCACAAAAATTTAAATAATTCATTACAAGCCTTTATTAAAAAAAATTTTCTCCCCAAAGTAAACAGACAGACAATGTCTAGTCTATTTGAAATGCCTGAAAGCAGAGGGGCTTCAAGGCAGTGGGAGAAGGTGCCTGTCCTCTGCTGGACATTTGACAACCAGCCCTTTGGATGGTTTGGATGTATAGGAGCGAAGGTGCAGACAGCAGTGGGGCTTAGAGTGGGGTCCTGAGGCTGTGCCGTGGCCTTTCTGGGGTTTAGCCACAATCCTGGCCTGACTCCAGGGCGAGGCAGGCCAAGGGGGTCTGCTACTGTGTCCTCCCACCCCTACCTGGGCTCCCATCCCCACAGCAGAGGAGAAAGAAGCCTGTCCTCCCCGAGGTCAGCTGCGTTAGAGGAAGAAGACTGGGCATGTCTGGGCAGAGATTTCCAGACTCTGAGCAGCCTGAGATGTCAGTAATTGTAGCTGCTCCAAGCCTGGGTTCTGTTTTTTAGTGGGATTTCTGTTCAGATGAACAATCCATCCTCTGCAATTTTTTAAAAGCAAAACTGCAAATGTTTCAGGCACAGAAAGGAGGCAAAGGTGAAGTCCAGGGGAGGTCAGGGGTGTGAGGTAGATGGGAGCGGATAGACACATCACTCATTTCTGTGTCTGTCAGAAGAACCAGTAGACACTTCCAGAATTGTCCTTTATTTATGTCATCTCCATAAACCATCTGCAAATGAGGGTTATTTGGCATTTTTGTCATTTTGGAGCCACAGAAATAAAGGATGACAAGCAGAGAGCCCCGGGCAGGAGGCAAAAGTCCTGTGTTCCAACTATAGTCATTTCTTTGCTGCATGATCTGAGTTAGGTCACCAGACTTCTCTGAGCCCCAGTTTCCCCAGCAGTGTATACGGGCTATGTGGGGAGTATTCAGGAGACAGACAACTCACTCGTCAAATCCTCCCCTTCCTGGCCAACAAAGCTGCTGCAACCACAGGGATTTCTTCTGTTCAGGTGAGTGTAGGGTGTAGGGAGATTGGTTCAATGTCCAATTCTTCTGTTTCCCTGGAGATCAGGTTGCCCTTTTTTGGTAGTCTCTCCAATTCCCTCCTTCCCGGAAGCATGTGACAATCAACAACTTTGTATACTTAAGTTCAGTGGACCTCAATTTCCTCATCTGTGAAATAAACGGGACTGAAAAATCATTCTGGCCTCAAGATGCTTTGTTGGGGTGTCTAGGTGCTCCAGGTGCTTCTGGGAGAGGTGACCTAGTGAGGGATCAGTGGGAATAGAGGTGATATTGTGGGGCTTTTCTGGAAATTGCAGAGAGGTGCATCGTTTTTATAATTTATGAATTTTTATGTATTAATGTCATCCTCCTGATCTTTTCAGCTGCATTGGGTAAATCCTTGCCTGCCAGAGTGGGTCAGCGGTGAGCCAGAAAGGGGGCTCATTCTAACAGTGCTGTGTCCTCCTGGAGAGTGCCAACTCATTCTCCAAGTAAAAAAAGCCAGATTTGTGGCTCACTTCGTGGGGAAATGTGTCCAGCGCACCAACGCAGGCGAGGGACTGGGGGAGGAGGGAAGTGCCCTCCTGCAGCACGCGAGGTTCCGGGACCGGCTGGCCTGCTGGAACTCGGCCAGGCTCAGCTGGCTCGGCGCTGGGCAGCCAGGAGCCTGGGCCCCGGGGAGGGCGGTCCCGGGCGGCGCGGTGGGCCGAGCGCGGGTCCCGCCTCCTTGAGGCGGGCCCGGGCGGGGCGGTTGTATATCAGGGCCGCGCTGAGCTGCGCCAGCTGAGGTGTGAGCAGCTGCCGAAGTCAGTTCCTTGTGGAGCCGGAGCTGGGCGCGGATTCGCCGAGGCACCGAGGCACTCAGAGGAGGTGAGAGAGCGGCGGCAGACAACAGGGGACCCCGGGCCGGCGGCCCAGAGCCGAGCCAAGCGTGCCCGCGTGTGTCCCTGCGTGTCCGCGAGGATGCGTGTTCGCGGGTGTGTGCTGCGTTCACAGGTGTTTCTGCGGCAGGTGAATGACGGGCGTGGGTCGGTGCGCGCTCGGCTTGCGCACACGGTGTCTCTAAGTGCGCGGGTGACGAGAGTCGGGATGTGCCGGAGACCCCGGGGCGGAGAGCGGGATTACAAGTACAGGAATCCCTGGTCACGCTCCCCGCCCCTGGAAACCCAGCTGGGGCGAGGGAGGGCGTGGACGGGACCGTTCTGGGAGCTCGCCTTTGGCTGCGGTTGGCTCCAGGCCCCAGGCGCAGTTTGCTCGCGGCGTGGGGATGAAGTCCGTGTCCCTGGAGGGGCCCAGGAAGGGCGAGGAAAGCGGAGTGGAGTAAGTTCGTCTAGGATCGGTCCCGGGTGGCTCTGGGATCCAATCTGCGCCGCCCTGGCCCAGGTCCCAGGTTCAGGTCCTTTACGCCACTGTGTCCACCACCTGGCTGAGCGCTGAGGTCAGCGCGGGCTGTTTCCTGGCCCTTGGGAATGTGCCAGGACCCGTCCCCTAAGGACTAGCGAGGAGGTGACTCACTGTGACAAGGAGACCCCAGGGAACGGACTGTATGAGGTCAGAACCCCGCCCGGGATGGGGTACAGCGGGACTCCAGAAGCCCTCTCCCCTGCCCCTTCGCGGTCTCCGTCCTCCCATCGGCACAGTGACCTATTTGGCTGGAACAGTTTGTTCCCAAGGAAGCCGGGCACTGGAGGTCCGGGACACCGCGTCGGGTCCCCGCTCCGCGGCGCGCTGTAGGGGTCGGGGAGTCACGGCCCTGCTCTGGGCGGGCTCTAACCAGCCTGTCAGTCGGGGAAGGGCAAGGGTCTCCTCTACCTCTTTCCCACCGCGGCCGGGAGAATCGCGGCCCAGCCTGTCCTCGGGTCGGGGCGCTGGACTCCGGGGCGGGAGCGGAGCCCACGCCTGGATGGGAGGCGGGGAGGGTTCATGTCTTTGAGGGGTGGGGGGTCTGGGGGGCACGACGCTGCTCAGGGCCTCTATCAGCTGCCTCGGGGGCTCAGGGCTTCCCGACCTAGCCCAGATTCCCTCTCCGAAAGCTACAGGGCTGAGCGGAGCAGGGGGGCGAGTCGCCCCCTGGGGCGCCGCCGCCTGGCGCGGACCACAGCGCGTCCTCTCCGTCCCAAACCCCTGGGGGACACTTGCGCCCTCTTCGTGAGGAAAAGCATCTTGGAGCTGGGTTAGGAACTTGGGGCGCCCAGGCAGCTTCCCCTCTCCTTGCCTCCCTCCACGTCGCGTTTCTGGGAGGACTTGCGAGCGGTTTTGTTTTCGTTGCTCCCGTCTATTTTTATTTTCCAGGGATCTGACTCATCCCGTGCTTTGGGCGTGGAGATAAGGTGGAGGGGCCGGCTCCCGGCGCGCGCGCGCGTGCGTGTCTGCGCGGGCGTGTGTGTGTGTGTGTGTGTGTGTGTGTGTGTGTGTGTCTGTGTCAGAGACGGCACAAGAGCGCGCGGTTTCCCAACAGCGGCGGGAGTTTCGGAAGCCTGGCCGGCTCAGCGTGACGTGTTCGCGGCCCCCCGGTCCCCTCCCATTCTCCCCCTCCCCACCCCAGGGTGACGCGCAGCCGGAGTGGAAGCAGTTTTGGCGGGCGAGCAGCGCCTTGCAGGAAACTGACTCATCACTACTCCCTCCAGCGGTCCGAGGCTCTGCCCACGCACCTCCCACTCCGCGCGTGATTTCCTGGAGGCCGGCGCCCCCTCCCGGCCCTGGCGGGAATAGCACACAGGCTTTCCCGCGGAGTGGGGCTGGCCGGCGCGAACCGCCGCGGCTACTCCTGGGCTCATCCGAGATCAACCCCTATGCCATTACCACCCCTTCAAAGGAGCACTCCTTAGGTTCAACAGTATTCACTGAGCTCTTACTGGAAATTAAAATATGGCTGAAGTCTAAGGCAGGAAGGCCAATAAAGGAGGCTATTTTTAATTGTTTCTAAAACAAGGGTTTGCGTTTCTGAGTTTTCTTTGGGCTGAAAGTTATTATGAGCATGAGAGCAGATTTTGATGGGGGAGGAGAGGCCTATGAGAGCCATAAGAGAAGGAGGGGTGGTAGAAGAGGAGAGGGTGCCTGCCTAGATCCTAGTCCTGTCTTGAACTCCCGAGAGCCAGGGAATATCCAGCACCTTGATGAAGCCCTAGGCGGGCGCCTCCTCCTTGTGCCTATGATGTATTGAGACCCAGAATGTCCATTTCAAACATACCAGTGTGTCTCCGCTTGGCTGGCAACCCAAGAGTGCCCATCTGAGGAATTGTGCCAAACACTTGCTTGAATCTTCAATCTGGATTAAGTTGGTCTCGGGAGGCAGGGCCTCAGCAATCTATATTTTGAAAAAACTCCCTAGGTGCTTTTCTTTCTTTCTTTTTTTCTTTCTTTCTTTCTTTCTTTCTTT

**>HepG2_BCL6_Peaks_66439:CDK14:7:90595839-90596619**

CCTTTCGGTAACCTCCCCACAGCCCAACACGCTGTCCCCAGACGCTGCCCGCTCCCACCCCGGTCCCTTCGTGATCCTCCCGCGCGCGGGCGGGGCGGGGGAGGAGCGCGGGGGCGGCCGGGAGGGGCGCGGCCGGGAGGGGGCGCCGCCGGGGCGCGAGCCAGACACAGAGGGCCGCGGAGGACGCGGGAGGCGAGCGGGGAGCCGAGGTCGTGCTAGGATCCCGCGCCCAGGGGCCTTCGTCGAGGGAGGGGCGTGGCGGCGGCAGCAGGAGGGCAGCAGGAGCTGTCAGGCTGGCGGTTCCGCGCCGCGGCTCCTTCTGCGCTGCGCCCGCAACTCTGGGCTGAGAACTTCTCCCGCCGGCCCACTCGCCCTCGCCCCGCGCACTCGGCGCCAGGCGACTCCCGCACCTTGTACCCGGCCCGCCCCGCCTCCTCCTTCGGCCGCACCACTCCCCCTGCCGGCCGCGCTTCTCTCCGTTACAAAGGAGGGAAAATGAGCCGGGCGGCGGCGGCGCGGCGCGGGGCCACCACGGCGGCGGCGAGCGCGGCCGCCCCCGGCACCACGTAAACCGCCCCCGCCCGCCCAGCTGCGGCCCAGGCCGGAGCGGAGCCTGCCGTCCTCCGCCTGCCTGCTGCTCGCCTCCCTAGACCTGCGCGTCGCTTCCCGGCCCGCCGAGGAGGTGGTGGAGGAGGAGGCGCCGCTTTCCCCGCGGCGCGCGCCCTCGCCGTTGTCTGAGCTGTGCCTGGACCAGTTTGGGGAAGTTGTCGGGGCTCCGCG

**>HepG2_H2A.Z_Peaks_2504:IGF1:12:102483959-102484898**

GAAAGCTCAGAATGGACATTTGGAGGTAAGGGAATTTTGGAATCAAATAAAGGGTGAGGCTTAAGGTTCTAGGGCAACCACCAGGCAAAGAACTTGGGGGAAACTATTGGATGTTTCTTTTGGATCATGGTTTGTGAGGAGGGTTGGTCATGGGTTGCTCAAACTAATGTTGGAGGTCAAGAACTTGTCAATTCAGAATATAAAACTCAGAGGAAAGACAGGGCCTCAAAAACTCAACATGCTGCCGAGCAAGGGGCCTAATGTCAGCTCCAAAACATCTGCTCAGAAAGATTCCTGTGATTCCCCAGGAAGATTTGCTCAAAGGAGCTGCTCTAACTCCTTAAAGAAGTGTTTCGCCCACGACTAGGGAAAACAGAAATAGGGAGGAGAAATCCTCTCACTAAAGAAATCAATGCTTTCTTTTCAACATCAAACTTGGATCTTATTTCTTCTAAAATGTCAAAAGGGCAAATACTGGAAACAGAAACGAAACTGTTGTGGTTGTGTGAGCAAAGTAATTCTTCGTTATGGCAGAGGAGAAGTCACGGCATGGGGTAAGGATGGGTTGGTGGAGTGAGAGAACTTGAGGACTCGTCACGCTTGCCCCACGTTGCATAAAATGTGGGGAAAAGAGACACCAACGCCAAACTTCAGGGGCAGGACCCACATAAATGCTTTCTTGGGGAATAAAAAAATGTGCGCGCCCTCTAGTGGACTTCTGAGAAACTGCCACTGCACTCTTGACCGTGGATGCGTTCTGGCAGGAGTGGGTTTTTTTTTTGAAAGCTCTGGATTTAAATACATTTTAAGCTACACTTATCTTTAGTTTTATTAAGTCTTTTGAAATAAGTAAGATAGATGTTCAACTCACTGTAGTTTAAAAAGGGGAAGATTGATAAGGGGGAAAGAAAGATCATAAACTTCATCAGGGCATGGATG

**>HepG2_H2A.Z_Peaks_3401:SPINT1:15:40856680-40860023**

ATTTGACTTGGTCTCTTCCATAGATTGGGGGTGGGTGTCAGAACCAGGCAGGCCCTGGGAGCCCCTTATTCTACCCCTTCTTCCCCCAGGCTCTGTGGAGATGGCTGTCGCAGTGTTCCTGGTCATCTGCATTGTGGTGGTGGTAGCCATCTTGGGTTACTGCTTCTTCAAGAACCAGAGAAAGGACTTCCACGGACACCACCACCACCCACCACCCACCCCTGCCAGCTCCACTGTCTCCACTACCGAGGACACGGAGCACCTGGTCTATAACCACACCACGCGGCCCCTCTGAGCCTGGGTCTCACCGGCTCTCACCTGGCCCTGCTTCCTGCTTGCCAAGGCAGAGGCCTGGGCTGGGAAAAACTTTGGAACCAGACTCTTGCCTGTTTCCCAGGCCCACTGTGCCTCAGAGACCAGGGCTCCAGCCCCTCTTGGAGAAGTCTCAGCTAAGCTCACGTCCTGAGAAAGCTCAAAGGTTTGGAAGGAGCAGAAAACCCTTGGGCCAGAAGTACCAGACTAGATGGACCTGCCTGCATAGGAGTTTGGAGGAAGTTGGAGTTTTGTTTCCTCTGTTCAAAGCTGCCTGTCCCTACCCCATGGTGCTAGGAAGAGGAGTGGGGTGGTGTCAGACCCTGGAGGCCCCAACCCTGTCCTCCCGAGCTCCTCTTCCATGCTGTGCGCCCAGGGCTGGGAGGAAGGACTTCCCTGTGTAGTTTGTGCTGTAAAGAGTTGCTTTTTGTTTATTTAATGCTGTGGCATGGGTGAAGAGGAGGGGAAGAGGCCTGTTTGGCCTCTCTATCCTCTCTTCCTCTTCCCCCAAGATTGAGCTCTCTGCCCTTGATCAGCCCCACCCTGGCCTAGACCAGCAGACAGAGCCAGGAGAAGCTCAGCTGCATTCCGCAGCCCCCACCCCCAAGGTTCTCCAACATCACAGCCCAGCCCGCCCACTGGGTAATAAAAGTGGTTTGTGGAGTTTCTGGCTCTGTTCTGTGGGCCAGGAGAGGGACTAGGTTGCTGGGGGGTGATGGGACCGTCCTGTTCAGCTGGTTCAGGCTTCAGCCACCCTGTTGAGCAGGTTTTCCCATCGTCTCTCTCGAAACTGGCCTGGCCCCTGACTTTGGAGAGCAGTGCCATGTTTGACCACCAGAGGGGAGGAGTGAGCGGGCCACGGGAGCCACGAGTGTGGGGAGGCTGCAGTGCTGCTGTGTAGCATTTCTTCCTGGTGTGAGCCTCTCCACAAGCCTGTTGAGTGGTTGAGCTCTCCACAATTCTGCTCATTTGGAGGTTGGGGTTAGGGGTGCACAGGCACCTTGGCTCCTACCTCCATGTTAGGGCCCAGGTGACTAGCTGTCTCCCTCCCAACAGAAGCCCTGGCTTCTGCGCTGGAGTGCAGCACCCCCAACCAGGCTCTGAGCTCCTTCCCAGGAGCCTTTAGTAGAGACACGCCACTCCAACCCACACCCCTGCATTTGTTCAAGCTACCTCCCGGTGCCAAAAAAAAAAAAAAATCATACTTTGAGCTCATTCGTTCGTTTATTCAGTGAATTACATTGAGCTCCTCTGTGCCAGACACTGCCAATCATTAGAAATTTGATACTGCTGAGAGGGGCAGAGGAAGCACTATTTAAATGCTTTACGTATCTCGAGTTGCTGGCGCTGCACACAGCCCTTGTGATGACAATACAATCATCCGCATTTTACAGGTGAAGAAACTAATTCATTGAAATTTCCTCCCCAAGGCCCTAGAGCTGAGATTGGACTGGATCCCCAGAGTTTAGTCTTACAAAAGAGGTATCGGGGTTGGTGGGGGTGGGGGGGGACTTAAAAGCAAATTTTAAGCAAGTACAACTACCATGAATGTACATATATGAAGTTATAAGTGTATAGCTCAGTGAATTTCCATAATGACACACACCTCTGAGGCAGGCACCCAGATCAAGAAGCAGAACCTTCTTAGCACGCCGGGACAGGCCTCGTTTTGATGGATAACACCTGGTTTTGGACTCGTGCTCTCATGGACCACCTCCCCATTTTGCACGCCTCAGCCTAAGCTCAAAACCCTCCCACACAACTGCAGGTGGTGGACACTCCCTGCCTCACACCCCTGTGTCTGCTAACACCACCCTGAGCCCAGAGCACCTCTTATTTCCTTTACTGCCTGTCTTCTTGCCGGATTGTGAGATCCCAGAGCCGCAGATGGCATCCAGTTGTGTCTGGGTTCCCGGCACCCCATGCTCCCTCTGTGACCTTGGTAAATCGTAGTTTAGTAAATGTTGAGTAAGCGGCAGGGGGTGGGGCTCATCCCCATTGTTCCACTTCATAGGGCACCGTGTTTTCCCTTCTAACCCCCTACTCTGACACTCGTCCATCCTTCGAGGCTCAAAACGGACTTGATCCCTACTGGGAACAACCCCCATTATCAGTTGGTTCTCAGACTCTACCCTAGTGTCCAGAACAGTGATCAACACAGAGCAAGTATTTAATAAGGGTTTGTTGGCCTGAAGTGAACATCCTCTCAGGGAGGGATAGACATCAAGTGAGAGGATGCCAGGCAAAGGGCCACCCCTAGTAACAGCTGCTTGCATGTGCAGAGGGAGTGCCCGAGGAGGTGGGAGCTCTCGGGGGTCACTAGGGGGCGCTGTGACTATGACTGGATGCCGTGTTCTTCCTGCAAGGATGTGAGGACTCAGTCTCAGGCAGGTGACAGGAGTGGAGCAATGAACGCCAAGACACAGCTCCTGCTCTCCTGGCGCTTACACTCTGGCGTGCAGGCTGCAGGGATGCAGATACGGTGACAAAACAGTCTGGTCCCCAAACTTCTCCTTATCCCTGAGACCGCCCCAGCCATCCTCTGCTCTGTGCCCACCCACATGACTCAGAACTTTGATCCCTACCTCCATGTCCTGAACAGGCAGTTTCCTCCACTTCAGAAGTCCCCTCCGCCCTGGAAAGCTCCTACTTTACCCCGTGTTCCAGCTCACGAAGCTTTCTCTGGCTCTCCAGCCAAAGTTCATTGCTGCCCTCTCCACGCACTCCTGCTCTACACAGCTCCGCTGCACGCATAAGTCCAAGCTAGTGTGTGTCTCCCTTTATCCAGACAAGACTCCTCAGGGCGCTGACCAGGTCTTAGTTATCCTAGCGTCTCCCAAGCTGGGCCCTGCTTGTGCGTACCAGGTATCTGAAAAATGGCTGCTGGAACAAAACAGAGGCCGGTCAAGTGGAGGAGATTAAGGTTAATAAGTGACTTCGTGGAGAAAGTCTAACATCAGGTGAGTGGCCCTGCACGGTGGCTCACACCTGTAATTCCAGCAGTTTGGGAGGCCGAGGTGGGTGGATCACT

**>HepG2_H2A.Z_Peaks_4538:JUP:17:41777010-41778389**

AAAAAGAAAAATAATAAAAATTTAAAAAGGCGAGGGCTGGCACAGAGACCTGTCGGGCAAGGTGGATGGCAGTCTGTCTGGGGGAGCCCTGGCTGCTTTCTCTGACCCCTGAGATGGGGACTAGGTAGGGAGCTGCCTCCCAGGCCCTGGGGAGGTCAAGGGAACATGGCTGAGGAGAAGGGGAGTGGAACTTCGCTCACGCCAGGTGGAGACACTGGCTGCCCTATGCCCAGTCCATGCTGCTTCCTGCAGCCAGGGGCAGCTGCACAGCCAGGCGTGAACATTCCCCACATAGCAGTCCCAGGGGGAAGCTGGGCTCAGCAGGATCTCCAGCCCCCACCTTCCCCACTCTGTCCCTGGCTCCTGCTGAGGTCTGGCCAGCAGTTCCAAGCACAGACACAGACTCTAGAAAGTCCAGAGAGATGGACATGATGGCTTCCCTTGAGGGCCTGTTTCCCTCTCAGGCTGGGGCTGCCCAAGGAGGCCTCTCCCCTCTGGCTGGCAGCTCCAGGACGGAACCTCCTGCAGACTACAGGTTCTCTGGCAGACACTGTGGTCTCCATCAGCAAGCTGGTCCATTCCAAGAGGGCAGAACTGAAGCAGGAGCTGTCAGACAAGCTGGTCTTAGGCTTCCGGAGGAAAACCCTCACTTCCAGTCTGGAGAGCAGCCCAGGCTGTGGCCTGAGGATGAGCTGGGGCCTGGGGGAGGCTCAGGCCTGCCGACCCAGCTGCTGTTTATTTTCCTCCTTCTGGGCTCCTTCCCAAAGCAAACAAACCCGGGTAGGAAAATGAAGGGGATTTGTAGGAGAGGGAATTTGCCACGGCCAAGTGGCTCGACCCAGGGACCTCTATGCTCCCCTGGAGAGGGCTCCAGCTGGAGCTCCGTTCCCTGCTGGTGACCCCCGTGGGCAGATTCCCAAACTGGCAGATGACCCCAAACCGGGACTCCTGGGAATGGAAAAACACTTCACCCCACACCAGGAAACAACAACAAAAGGTTGACGTGTGTTGGTGACGCAAAATACACAAGTACTCTCATTCCACTGCCTCCAACCACCACCAGTGGGGCTTGGGCAGCCTGGGGTCCAGGGGCGGCCCACTTCTCAATAACAGCAGCTCCGATCCCAGCTGACTGAGGCCCACTCCCTGGGGTGTGATTCTGCAAGGATCCCTACATATAACCCTCTCCTGGGAAGATCCATCTGTTTATTGTACAACACATGCTGGAAAACCAGGATTGGAATAAAGTTTTGGCTAAAATCTGTTACTGTAGGCAGGGTGCAGTGGCTCACCCCTGTAATCCCAGCACTTTAGGAGGCCAAGGCAGGTGAATCCCCTTGAGCTCAGGAGCTCCAGACCAGTCTGGGCAACATGGCGAA

**>HepG2_H2A.Z_Peaks_5173:TCF:19:1652475-1653706**

CCGTGCGCGCGGCCGGCCGGGGCGCCCCTGGGGCAGCGGCGTGCGCGGTGCCCGCGGTGCCCGCCGCCGCGTCGGCTCCGGCCCGCTACGCCCGCAGCCGCCGCCGCTGCCTCATCTTCCTGCGGCGGGAGACATGTTCCGCCCCCCGCCCGCGCCGCCCCGCCCCGCCCCGTGCAGGCCCCGCCCCTGCCCCGCCCCCGAGTGCCCCGCCCGGCGGCCCACGCGGATCCCTCCGCCACCTCCGGGCGGCTCGGGCCCGAACGCCCTAGCTCGGCCTCTCGAGCACCCTCGTGAGGACCCCGAATCCCGTGAGGTCCAGAGCCTGGGAGCGGGGACGCGCAGAGGAGGCGGTCGGGCCACGGCGCGGGGAGAAATCACGGACTATCCCCGTCCGCGGAAGCACACACGAGCTGTGCGCTTAGTCCATGACGCAAGCGAGTAAGGCCCGTGAGATTGAAAGCTAAGGCAGAGCAGTCTGGTCAATCGGAAGCCGCGAAAGTCTGATGGGCGGGGATCCTAGCCATTCGTGGTGAGGCCCCGCCTCCTTTCTTCTCGGCCCCGCCCCTCAGCAGAGGCGGGACTCTGCGAGCGAGAGGCCGCGAGAGGCGGCCGGGGTGGGTCCTGGAGTTTTGTTCTCAGGTTGGCGTGGCCGCCCGCGCGGAGCCTTCTGCTTGGTTATATTTGCGTTCCTCGGGCCGGCCCCGCGTGCTGAGTGGTGCGAGCGGGTATCACGGCCCCGAGGGGGCTACGTCAGACCCATTTTCCCGGCGGGAAAACCGACTCTGGCTCCGGCTCTGGGTCAGAGAGCCCCGCGGGGAGTCTCAGCGGTGCCTTTGCCGACTTGAGTCTCCGTCTCGGCATCTGTGAATTTGGACCTGACTTTGGACAGAACTCAGGCCAGATCTGGCTTCCTGATTCTTAGTAGTCCGTGTCTACATTTTAGTAAAAAGTGACCCCGCCTAGGATCGGGCGCCGTGGCTCCTGCCTGTGATCCCAGCGCTTTGAAGGAGGCCGAGGCGGGAAGATCGCTTGAGGCCAGGAGTTCGAGAACAGCCTGAACAACATAGTGAAACCCCCCCCACCCACACCCGTCTCTAAAAATATTTAAAATAATTTAAAATAATAATTTAAAAAGTTGACCGGACGCGGTGGCCCACGCTTGTATTCCCAGCACTTTGGGAGGCCAAGGCGGGCAGATCACCTGAAGTCAGGAGTTCGAGACCTCTCTGGC

**>HepG2_H2A.Z_Peaks_9620:CDKN1A:6:36675728-36677847**

CGCCCGGCCAGTATATATTTTTAATTGAGAAGCAAAATTGTACTTCAGATTTGTGATGCTAGGAACATGAGCAAACTGAAAATTACTAACCACTTGTCAGAAACAATAAATCCAACTTTTTGTGCAAAAAAAAAAATACAAATATTAGCTGGGCATGGTGGTGCATGCCTGTAATCCCAGCTACTCGGGAGGCTGAGGCAGAATTGCTTGAACCTGGGAGGCGGAGACTGCAGTGAGCTGAGATTGTGCCACTGCTGACTTTGTCTCAAAAAACAAAACAAAACAAAAAAACAAAATGAAAACAAAAAGCCAGGGCTGCCTCTGCTCAATAATGTTCTATCTTTGTTCCGCCTCTTCTCTGGGGTCTCACTTCTTGGGAGCCTGTGTGAAGGTGAATTCCTCTGAAAGCTGACTGCCCCTATTTGGGACTCCCCAGTCTCTTTCTGAGAAATGGTGACATTGTTCCCAGCACTTCCTCTCCCTTCCTAGGCAGCTTCTGCAGCCACCACTGAGCCTTCCTCACATCCTCCTTCTTCAGGCTTGGGCTTTCCACCTTTCACCATTCCCCTACCCCATGCTGCTCCACCGCACTCTGGGGAGGGGGCTGGACTGGGCACTCTTGTCCCCCAGGCTGAGCCTCCCTCCATCCCTATGCTGCCTGCTTCCCAGGAACATGCTTGGGCAGCAGGCTGTGGCTCTGATTGGCTTTCTGGCCGTCAGGAACATGTCCCAACATGTTGAGCTCTGGCATAGAAGAGGCTGGTGGCTATTTTGTCCTTGGGCTGCCTGTTTTCAGGTGAGGAAGGGGATGGTAGGAGACAGGAGACCTCTAAAGACCCCAGGTAAACCTTAGCCTGTTACTCTGAACAGGGTATGTGATCTGCCAGCAGATCCTTGCGACAGGGCTGGGATCTGATGCATGTGTGCTTGTGTGAGTGTGTGCTGGGAGTCAGATTCTGTGTGTGACTTTTAACAGCCTGCTCCCTTGCCTTTTTCAGGGCAGAAGTCCTCCCTTAGAGTGTGTCTGGGTACACATTCAAGTGCATGGTTGCAAACTTTTTTTTTTAAAGCACTGAATAGTACTAGACACTTAGTAGGTACTTAAGAAATATTGAATGTCGTGGTGGTGGTGAGCTAGAAGTTATAAAAAAAATTCTTTCCCAAAAACAACAACAAAAAGAATTATTTCATTGTGAAGCTCAGTACCACAAAAATTTAAATAATTCATTACAAGCCTTTATTAAAAAAAATTTTCTCCCCAAAGTAAACAGACAGACAATGTCTAGTCTATTTGAAATGCCTGAAAGCAGAGGGGCTTCAAGGCAGTGGGAGAAGGTGCCTGTCCTCTGCTGGACATTTGACAACCAGCCCTTTGGATGGTTTGGATGTATAGGAGCGAAGGTGCAGACAGCAGTGGGGCTTAGAGTGGGGTCCTGAGGCTGTGCCGTGGCCTTTCTGGGGTTTAGCCACAATCCTGGCCTGACTCCAGGGCGAGGCAGGCCAAGGGGGTCTGCTACTGTGTCCTCCCACCCCTACCTGGGCTCCCATCCCCACAGCAGAGGAGAAAGAAGCCTGTCCTCCCCGAGGTCAGCTGCGTTAGAGGAAGAAGACTGGGCATGTCTGGGCAGAGATTTCCAGACTCTGAGCAGCCTGAGATGTCAGTAATTGTAGCTGCTCCAAGCCTGGGTTCTGTTTTTTAGTGGGATTTCTGTTCAGATGAACAATCCATCCTCTGCAATTTTTTAAAAGCAAAACTGCAAATGTTTCAGGCACAGAAAGGAGGCAAAGGTGAAGTCCAGGGGAGGTCAGGGGTGTGAGGTAGATGGGAGCGGATAGACACATCACTCATTTCTGTGTCTGTCAGAAGAACCAGTAGACACTTCCAGAATTGTCCTTTATTTATGTCATCTCCATAAACCATCTGCAAATGAGGGTTATTTGGCATTTTTGTCATTTTGGAGCCACAGAAATAAAGGATGACAAGCAGAGAGCCCCGGGCAGGAGGCAAAAGTCCTGTGTTCCAACTATAGTCATTTCTTTGCTGCATGATCTGAGTTAGGTCACCAGACTTCTCTGAGCCCCAGTTTCCCCAGCAGTGTATACGGGCTATGTGGGGAGT

**>HepG2_H2A.Z_Peaks_10335:CDK14:7:90462446-90464307**

AGGAGAAGCAAGAGAGCAAACATGATTTATGAAAAAAAGGAGCTTTATACACGGGATTTGTAGACTCACATTGTCCCCTTCCTCATTGCGGATATTGAGATCAACTCCAGGAAAGGGCATGAGAACCTTCCTTAAGAACAATAATTGCAAATCTGTTACCATCATCTTTCCTGCCTATCAGGAGTCTTAATATGTTACTGTCAAGGGATGCTCAAGAGACAGAAACCATAACTCAAACTGGCTTAAACCAATGGTGAATTTATTGACTTGGATAGTGATAATGGAACAACCTGACTCCAGGCATGATCTGATCCAGTGTCCAGATGGATCACCAGAACCTGTTTCTTTTTCTGAAACAACTCCATTTTCATGCTGGCTCTCTCCTTATGAGGTGGGGTTTTCTGAGAAGCAGATGCCAAGACAGGATTAGATATGGTTATGGTTTGGATCTCCATGTATCAATGCAACTCAGGCCCTGTGTCCAGTAATTTTTAAAATGTCAAGTATTCCACTTTTCCTGGTGTACAGACCCCCTAGTAAGTGGTGGTAAGGACTGGGAGAATTATAGCATATATTTGTAGCAATATTGCAGAGTCCCTTCTAGGAACCTGGCCACCTCTTCATTCAATGGGTCTGGATCTGAAAATTAAATAAAGTTTGGGAAGTGAGTGATATGGTTTGGCTGTATCCCCACTTAAATCTTATCTTGACTTGTAGTTCCCATAATCCCCACGTGTTGTGGGAGGGACCCAGTGGGAGGTAATTAAATCATGTGGTGGTTATCCCAAGTGCTGCTGTTCTCATGATAGTGAGTAAGTTATTACAAGATCTGATGGTTTTATAAAGGGTTATTCCTTCTTTGCTCAGCACTTCTTCCTGCTGCCATGTGAAGAAGAACATATTTGCTTCCCCTTCTGCCATAACTGTAAGTTGAGGCTTCCCCAGCCATGTGGAACTGTGAGTCAATTAAACCACTTTCCTTTATAAATTACCCAGTCTCAGATATGTCCTTACAGCAGCGTGAGAATGGACTAATATAGTAAATTGGTACCGGGTAGTGAGGTACTGCTCACTAGCCGGTGAAAGTGAAATGTGAAAGTGAAAGTGAAAATGTGAAAGTGACTTTGGAACTAGGTAACAGGCAGAGGTTGGAAAAGTTTGGAGGCTCAGAAGAAGAGAGGAAGATGTGGGAAAGTTTGGAACTTCCTAGAGACTTGTTGAATGACTTTGACCAAAATGCTGATAGTCATAGGGACAGTGAAGTCCAGGCTGAAAATGGAGATTAGGAACTTGTTGGTAACTGGCATAAAGGTGACTCTTTCTATGTTTTAGCAAAGAGACGTGGCATTTTGCCCCTGCCCTAGAGATCTGTGGAATTTTAACTTGAGAGAAATAATTTAGAGTATCCAGTGGAAGAAATTTCTAAGCAACAAAGCATTCAAGAGGTGTCTTGGGTGCTGTTAAAAGCATTCAGTTTTACGTATTCACAAAGATTGGTTTGGAATTGAAACTTATGTATAAAAGGGAAGCAGATCATGAAAATTCAGAATATTTGCAGCCTGACAATGTGATAGAAAAGAAAAACCCATGTTCTTTCGGAGGAGAAATTCAAGCTGGCTTCAGAAATTTGCATAAGTAATGAGGAGCCAAATGTTGATCACCAAGATAATGGGGGAAAATGTCTCCAGCACATATCAGAGGTCTTCATGGCAGCCCCTCCCATCACAGGCCCAGAGGCCTAGGAGGAAAAAGTGGTTTTGTTGACTAGGCCCAGGGCCTTGCTGCTTTGTGCAGTCTAGAGACATGGTGCCCTGCATCCCAGCTGTGGCTAAAAGGGGCCAATGTAGAGCTCAGGCTGGTT
