# Supplementary figures and images for: H2A.Z acetylation by lincZNF337-AS1 via KAT5 implicated in the transcriptional misregulation in cancer signaling pathway in hepatocellular carcinoma
Source: Cell Death Dis. 2021 Jun 12;12(6):609. doi: 10.1038/s41419-021-03895-2 (PMC8197763; doi:10.1038/s41419-021-03895-2)

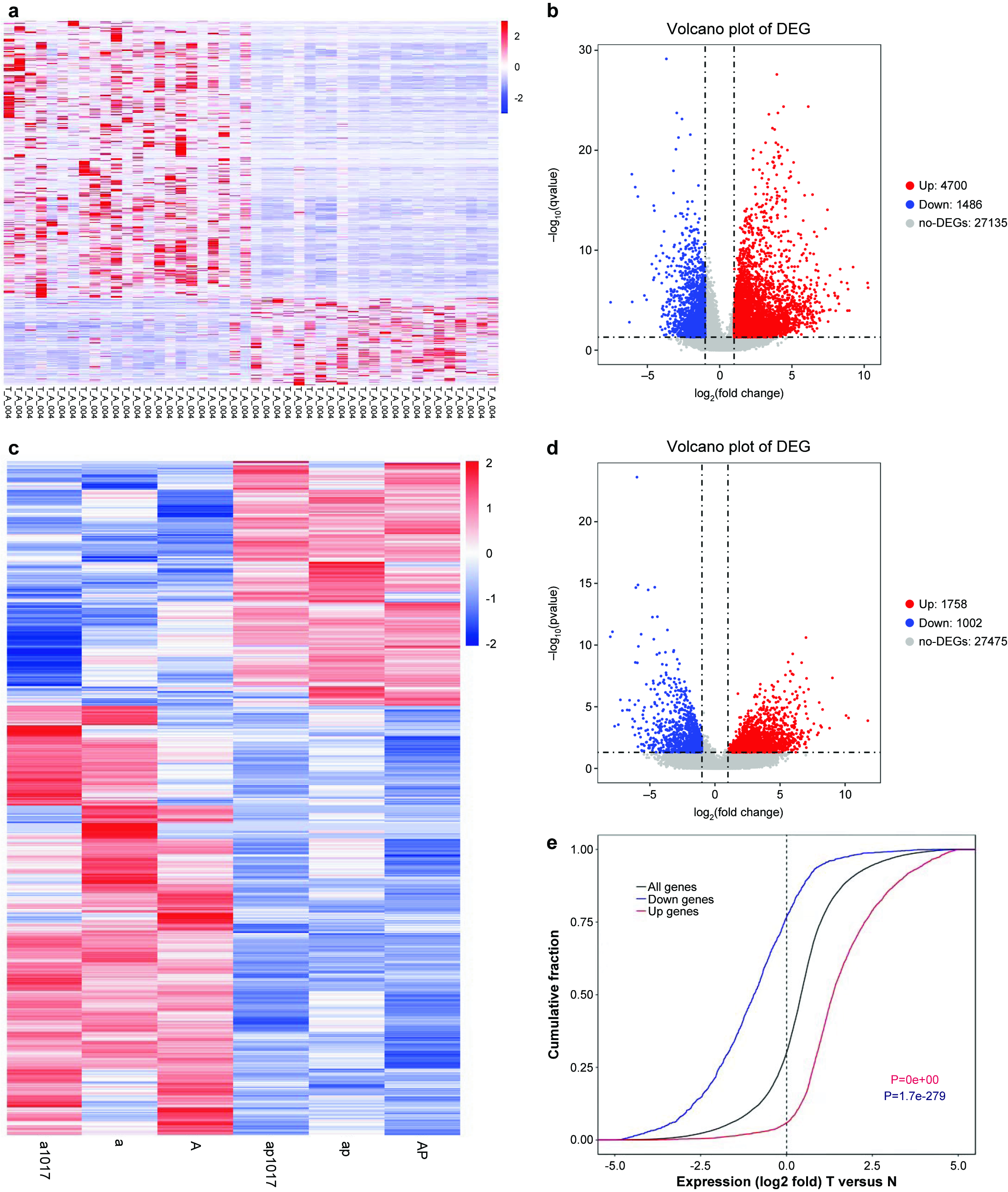

Supplement: Supplementary file 10 — Figure S1 [file 41419_2021_3895_MOESM10_ESM.tif]

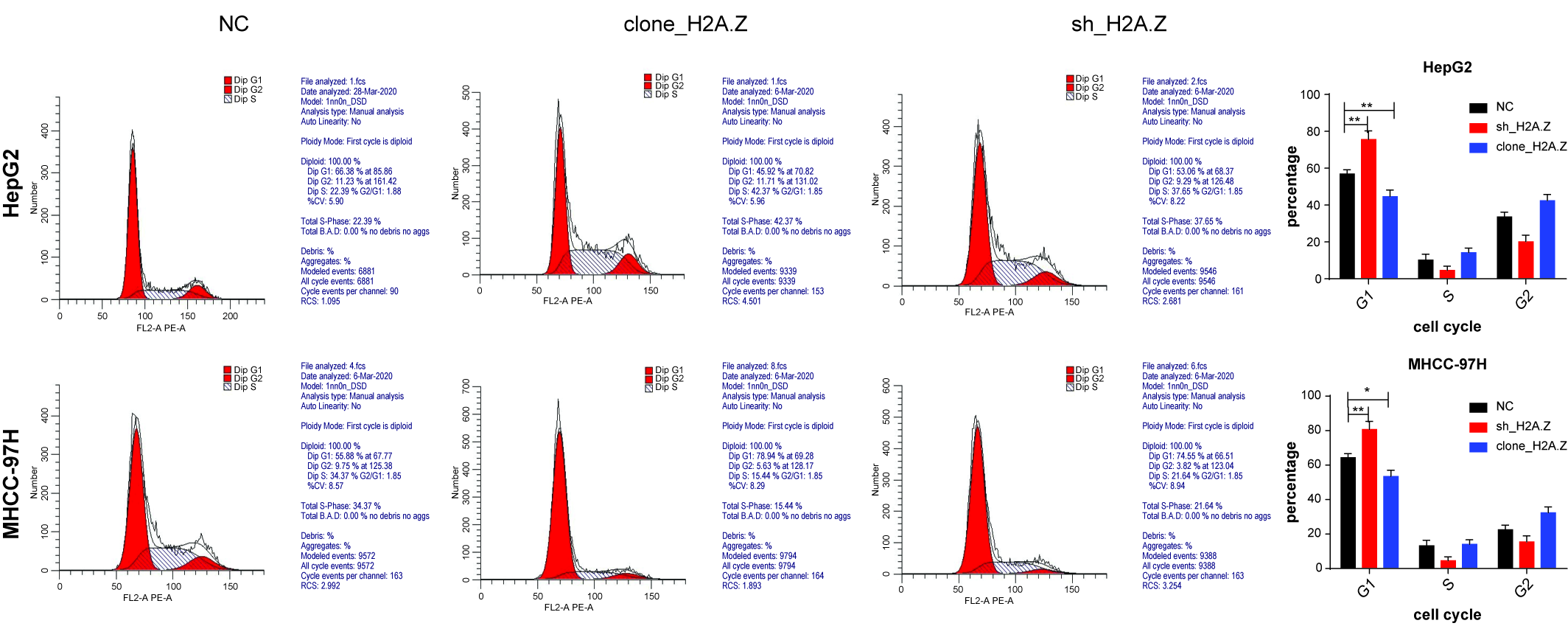

Supplement: Supplementary file 11 — Figure S2 [file 41419_2021_3895_MOESM11_ESM.tif]

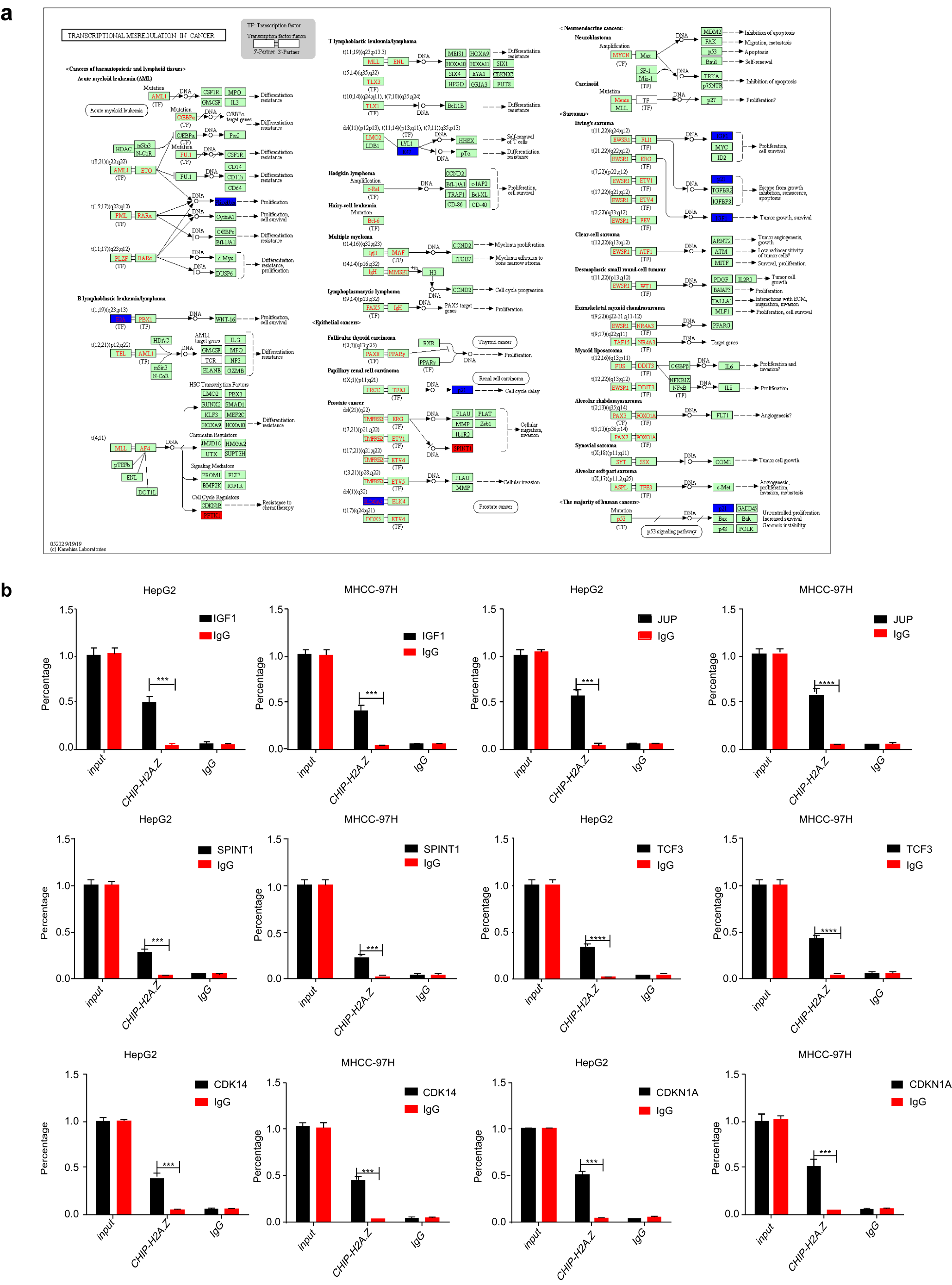

Supplement: Supplementary file 12 — Figure S3 [file 41419_2021_3895_MOESM12_ESM.tif]

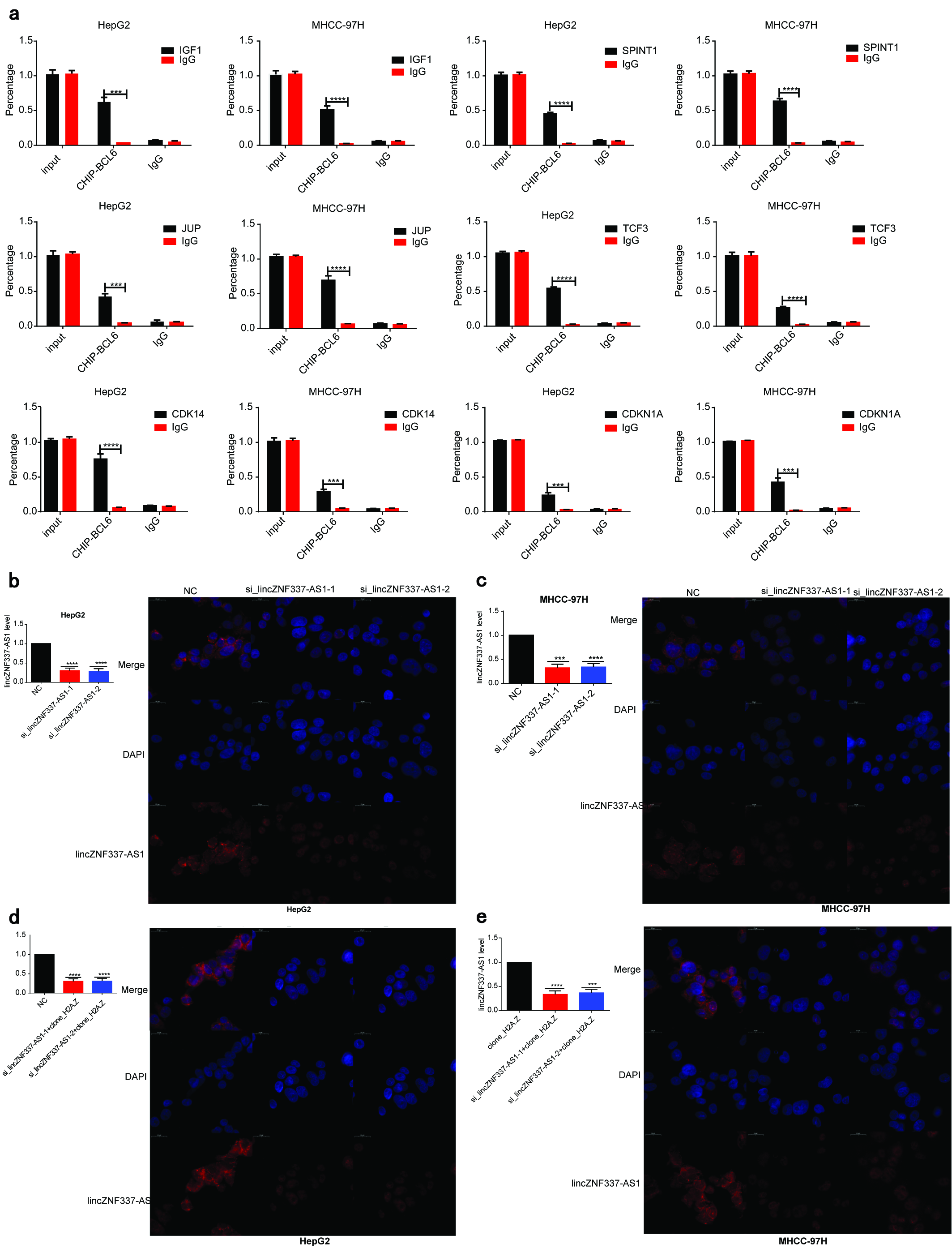

Supplement: Supplementary file 13 — Figure S4 [file 41419_2021_3895_MOESM13_ESM.tif]

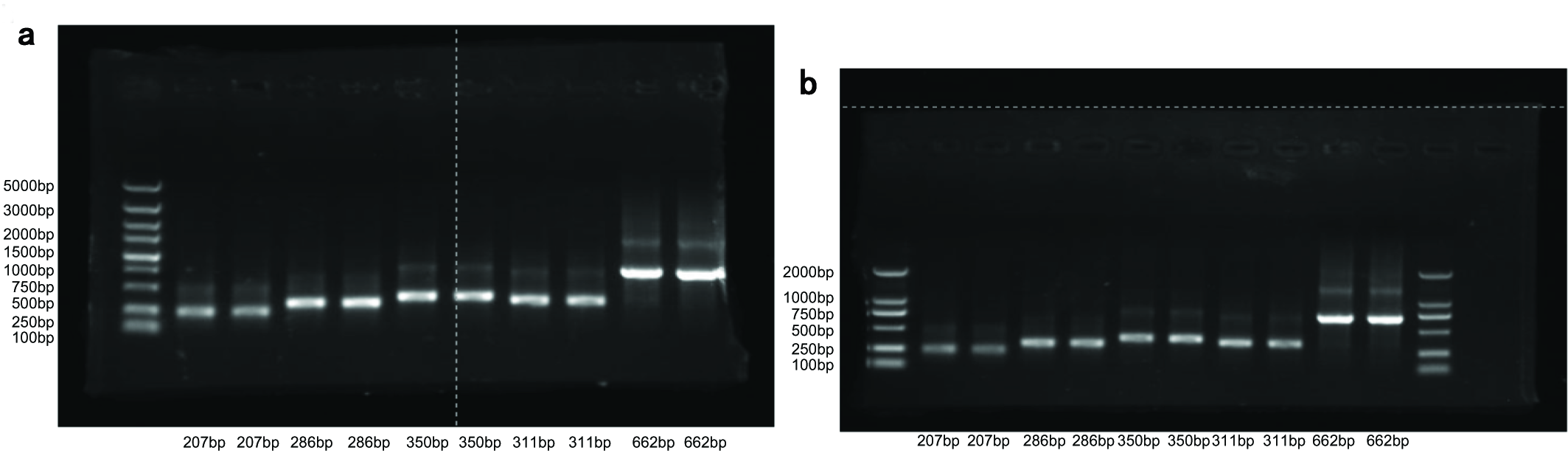

Supplement: Supplementary file 14 — Figure S5 [file 41419_2021_3895_MOESM14_ESM.tif]
