# Supplementary material for: H2A.Z acetylation by lincZNF337-AS1 via KAT5 implicated in the transcriptional misregulation in cancer signaling pathway in hepatocellular carcinoma
Source: Cell Death Dis. 2021 Jun 12;12(6):609. doi: 10.1038/s41419-021-03895-2 (PMC8197763; doi:10.1038/s41419-021-03895-2)
Supplement: Supplementary file 15 — Supplementary figures legends [file 41419_2021_3895_MOESM15_ESM.docx]

Fig.S1 Bioinformatics data analysis. a-b Heat maps and volcanograms of 23 pairs of HCC and paired paracancer tissues analyzed using RNA-seq in the GEO database(Software: R pheatmap and ggplot2, R package). c-d Heat maps and volcanograms of the three pairs of HCC and paired paracancer tissues analyzed by RNA-seq(Software: R pheatmap and ggplot2, R package). e Graphical comparison of the two groups of gene transcriptome data analyzed using RNA-seq, smooth curve, indicating high consistency of data(Software: ggplot2, R package).

Fig.S2 Flow cytometry analysis was conducted for detecting cell cycle. The data are expressed in terms of mean ± SD (Student’s t-test; * P < 0.05; ** P < 0.01; n=3 in each group).

Fig.S3 Verification of downstream target genes of H2A.Z. a Schematic diagram of transcriptional misregulation in cancer signaling pathway, E47 synonymsTCF3; p21 synonyms CDKN1A; PFTK1 synonyms CDK14; plakoglobin synonyms JUP. b Chip-qPCR assay identify the downstream target genes of H2A.Z. The data are expressed in terms of mean ± SD (Student’s t-test; *** P < 0.001; **** P < 0.0001; n=3 in each group).

Fig.S4 Verification of downstream target genes of BCL6 and knockdown identification of lincZNF337-AS1 in vitro. a Chip-qPCR assay identify the downstream target genes of H2A.Z. b-e Identification of lincZNF337-AS1 gene knockdown effect by qRT-PCR and ISH in HCC cell lines and H2A.Z overexpression HCC cell lines(Scale bars: 20μm). The data are expressed in terms of mean ± SD (Student’s t-test; *** P < 0.001; **** P < 0.0001; n=3 in each group).

Fig.S5 Analysis of the in vitro transcription products. a Electrophoretic images of PCR products after plasmid amplification, the Maker strip size from top to bottom is in order: 5K, 3K, 2k, 1.5K, 1K, 750bp, 500bp, 250bp, 100bp. b Electrophoretogram of transcription products in vitro, Maker strip size from top to bottom: 2k 1K 750bp 500bp 250bp 100bp. (n=3 in each group).
